# Mitochondrial resilience and antioxidant defence against HIV-1: unveiling the power of *Asparagus racemosus* extracts and Shatavarin IV

**DOI:** 10.3389/fmicb.2024.1475457

**Published:** 2024-10-23

**Authors:** Pratiksha Jadaun, R. Harshithkumar, Chandrabhan Seniya, Shraddha Y. Gaikwad, Shubhangi P. Bhoite, Madhuri Chandane-Tak, Swapnil Borse, Preeti Chavan-Gautam, Girish Tillu, Anupam Mukherjee

**Affiliations:** ^1^Division of Virology, ICMR – National Institute of Translational Virology and AIDS Research, Pune, India; ^2^School of Biosciences, Engineering and Technology, VIT Bhopal University, Bhopal, India; ^3^CSIR-National Chemical Laboratory, Pune, India; ^4^AYUSH-Center of Excellence, CCIH-Interdisciplinary School of Health Sciences, Savitribai Phule Pune University, Pune, India

**Keywords:** HIV-1, *Asparagus racemosus*, Shatavarin IV, Ayurveda, antiviral activity, ROS scavenging, mitochondrial dysfunction, alternative therapy

## Abstract

*Asparagusracemosus* (AR), an Ayurvedic botanical, possesses various biological characteristics, yet its impact on HIV-1 replication remains to be elucidated. This study aimed to investigate the inhibitory effects of AR root extracts and its principal bioactive molecule, Shatavarin IV (Shatavarin), on HIV-1 replication and their role in mitigating mitochondrial dysfunction during HIV-1 infection, utilizing both *in vitro* and *in silico* methodologies. The cytotoxicity of the extracts was evaluated using MTT and ATPlite assays. *In vitro* anti-HIV-1 activity was assessed in TZM-bl cells against X4 and R5 subtypes, and confirmed in peripheral blood mononuclear cells using HIV-1 p24 antigen capture ELISA and viral copy number assessment. Mechanistic insights were obtained through enzymatic assays targeting HIV-1 Integrase, Protease and Reverse Transcriptase. Shatavarin’s activity was also validated via viral copy number and p24 antigen capture assays, along with molecular interaction studies against key HIV-1 replication enzymes. HIV-1 induced mitochondrial dysfunction was evaluated by detecting mitochondrial reactive oxygen species (ROS), calcium accumulation, mitochondrial potential, and caspase activity within the infected cells. Non-cytotoxic concentrations of both aqueous and hydroalcoholic extracts derived from *Asparagus racemosus* roots displayed dose-dependent inhibition of HIV-1 replication. Notably, the hydroalcoholic extract exhibited superior Reverse Transcriptase activity, complemented by moderate activity observed in the Protease assay. Molecular interaction studies revealed that Shatavarin IV, the key bioactive constituent of AR, formed hydrogen bonds within the active binding pocket site residues crucial for HIV replication enzyme catalysis, suggesting its potential in attenuating HIV-1 infection. Mitochondrial dysfunction induced by HIV-1 infection, marked by increased oxidative stress, mitochondrial calcium overload, loss of mitochondrial membrane potential, and elevated caspase activity, was effectively mitigated by treatment with AR extracts and Shatavarin IV. These findings underscore the potential of AR extracts and Shatavarin IV as antiviral agents, while enhancing mitochondrial function during HIV-1 infection. In conclusion, *Asparagus racemosus* extracts, particularly Shatavarin IV, demonstrate promising inhibitory effects against HIV-1 replication while concurrently ameliorating mitochondrial dysfunction induced by the virus. These findings suggest the therapeutic potential of AR extracts and Shatavarin in combating HIV-1 infection and improving mitochondrial health.

## 1 Introduction

Human Immunodeficiency Virus type 1 (HIV-1) remains a significant global health concern, with approximately 39 million individuals affected and 1.3 million new infections annually, posing a persistent threat to public health according to the Joint United Nations Program on HIV/AIDS (UNAIDS, 2023). Although antiretroviral therapies have significantly prolonged the lives of those infected, the need for lifelong treatment to prevent viral relapse, coupled with emerging drug-resistant strains and adverse effects, underscores the ongoing challenges in managing HIV-1 infection (Eholié et al., 2012; Deeks et al., 2015).

In the search for effective HIV-1 management strategies, natural products have gained significant attention due to their historical importance in drug discovery (Dias et al., 2012; Mandal et al., 2020; Serna-Arbeláez et al., 2021; Jadaun et al., 2023a,b). Approximately 42% of all drugs launched between 1981 and 2019 were derived from natural sources, primarily plants (Newman and Cragg, 2020). Within the domain of natural medicine, Ayurveda, an ancient Indian medicinal system, has long utilized botanicals for managing various health conditions. Several medicinal plants have been studied for their potential against HIV-1 infection (Kumari et al., 2015; Yan et al., 2016; Sanna et al., 2018, 2023; Sillapachaiyaporn et al., 2019, 2021; Omoruyi et al., 2020; Guzzo et al., 2021; Sistani et al., 2021; Bektaş et al., 2022; Messi et al., 2022; Jadaun et al., 2023a; Zhang et al., 2023). For instance, *Phyllanthus amarus* has been explored for its antiviral properties, particularly its ability to inhibit HIV by targeting the reverse transcriptase enzyme, a critical component of HIV replication, whereas, studies on *Phyllanthus urinaria* suggest its ability to prevent HIV-1 infection by binding to key viral proteins, such as HIV-1 RT, gp120, and P24 (Zhang et al., 2017; Ahmad et al., 2021; Saini et al., 2022). Similarly, extracts from *Tinospora cardifolia* leaves have shown promising anti-HIV-1 activity by significantly inhibiting the reverse transcriptase enzyme, exhibiting effects comparable to standard antiviral drugs (Estari et al., 2012). Leaf extracts from *Carica papaya* and *Psidium guajava* have demonstrated dual antiviral and cytoprotective antioxidant properties, which hold potential for novel anti-HIV-1 therapies or as complementary agents to current antiretroviral therapies (Jadaun et al., 2023b). Additionally, curcumin has been identified as an inhibitor of key enzymes like HIV-1 protease and integrase, while also blocking the NF-κB pathway, which is crucial for HIV-1 gene expression (Ali and Banerjea, 2016). *Terminalia paniculata* fruit extracts have shown strong anti-HIV-1 activity *in vitro*, with acetone and methanol extracts inhibiting reverse transcriptase by over 77.7% and protease by over 69.9% (Durge et al., 2017). Among the botanicals regarded in Ayurveda, *Asparagus racemosus* Willd. (AR), commonly known as Shatavari, holds a prominent place. AR is revered for its immunomodulatory and adaptogenic properties, making it a subject of growing interest in pharmacological research (Lele, 2010; Balasubramani et al., 2011; Patwardhan, 2014).

Numerous pharmacological activities have been attributed to AR, including galactagogic, anti-aging, nootropic, and anti-inflammatory effects, with a particular emphasis on its immunomodulatory properties (Alok et al., 2013). Previous studies have documented various bioactivities of both Aqueous (AQAR) and Hydroalcoholic (HAAR) extracts of AR (Gautam et al., 2004; Saggam et al., 2022). Notably, AR aqueous root extract has demonstrated immunoadjuvant potential, enhancing immunoprotection against live *B. pertussis* (Gautam et al., 2004), while another study suggests its role as a therapeutic adjuvant by modulating cytokines to prevent PTX-induced myelosuppression and associated morbidity signs (Saggam et al., 2022). These findings underscore the therapeutic potential of AR extracts with tangible clinical benefits. Despite the comprehensive documentation of the phytochemical profile and composition of AR root extracts (Saran et al., 2020; Borse et al., 2021; Guo et al., 2022; Saggam et al., 2022; Prasad et al., 2024), the exploration of AR and its constituents’ potential in combating HIV-1 infection remains largely uncharted territory.

HIV-1 infection is known to induce mitochondrial dysfunction through increased production of reactive oxygen species (ROS), aggravating oxidative stress in the host environment (Ivanov et al., 2016). Interestingly, AR possesses antioxidant properties, as evidenced by previous studies (Wiboonpun et al., 2004; Karuna et al., 2018). Furthermore, the protective role of AR extracts against ROS-induced oxidative damage in a rat liver mitochondrion model has been demonstrated (Kamat et al., 2000). Given these observations, we sought to investigate the anti-HIV-1 efficacy of AR extracts and their potential in mitigating ROS-induced mitochondrial dysfunction, leveraging their established safety profile, metabolite richness, and confirmed bioactivities. Furthermore, consistent with our current findings, previous studies have also identified Shatavarin IV as a prominent bioactive component of AR root extracts, renowned for its therapeutic potential (Mitra et al., 2012; Smita et al., 2017; Borse et al., 2021).

Therefore, this study sought to evaluate the anti-HIV-1 efficacy of aqueous and hydroalcoholic extracts of *Asparagus racemosus* roots, along with Shatavarin IV, using *in vitro* and *in silico* assays. Furthermore, we investigated the impact of these extracts on HIV-1-induced mitochondrial dysfunction, focusing on oxidative stress, calcium homeostasis, and caspase activation, with the aim of expanding our understanding of AR’s therapeutic potential in combating HIV-1 and addressing associated mitochondrial dysfunction.

## 2 Materials and methods

### 2.1 Plant material extraction and identification of Shatavarin IV

Roots of *Asparagus racemosus* Willd. were collected from authenticated sources and graded accordingly. Voucher specimens were deposited as *Asparagus racemosus* Willd. (AMAR-1) at the Botanical Survey of India, Koregaon Road, Pune, and authenticated with certificate number No.BSI/VWRC/lden.Cer./2024/0901240015667. The phytoextraction processes, with minor adjustments to established protocols, were utilized for the hydroalcoholic (HAAR) and aqueous (AQAR) extraction of *Asparagus racemosus* (Rodríguez-García et al., 2019; Huei et al., 2020; Somaida et al., 2020; Borse et al., 2021; El-Desouky, 2021; Jadaun et al., 2023b). Briefly, the roots of *Asparagus racemosus* were first minced using a mortar and pestle, followed by further grinding with a mixer to obtain a fine powdered consistency. For the hydroalcoholic extract of *Asparagus racemosus* roots, powdered raw materials were soaked in a 70:30 water-alcohol mixture at a 1:4 ratio (material) overnight. The extraction process was conducted for 3 h at a temperature of 60 ± 5°C. The resulting extract was filtered through a 400-micron strainer three times to ensure purity. The filtered extract was then pooled, concentrated, and subjected to spray drying to obtain a final dry powder. For the aqueous extract, crushed *Asparagus racemosus* root powder was macerated in 3 liters of UV-filtered water for 8 h overnight. After maceration, the mixture was gently boiled for up to 8 h to reduce the volume to 1/8th of its original amount. The resulting concentrated decoction was then filtered using a cotton cloth to remove any solid residues. Finally, the filtrate was lyophilized to obtain a dry powder extract. To prepare stock solutions for both AQAR and HAAR, the aqueous extract was dissolved in water, while the hydroalcoholic extract was dissolved in DMSO. For subsequent assays, the stock solutions were diluted in complete medium or PBS. Care was taken to ensure that the final concentrations of alcohol and DMSO were minimal and maintained within non-toxic levels for the cells. The presence of Shatavarin IV in the root extracts HAAR and AQAR was previously confirmed by our research group (Borse et al., 2021), and reaffirmed in the current study using a High-Resolution Mass Spectrometry (HR-MS). HR-MS (ESI) analysis revealed peaks at m/z calcd for C_45_H_74_O_17_ [M + H] + 887.4990 for AQAR and m/z calcd for C_45_H_74_O_17_ [M + H] + 887.5001 for HAAR, with corresponding found values of 887.4999 for both extracts. These results further confirmed the presence of Shatavarin IV in both AQAR and HAAR extracts (Supplementary Figure S1). The reference pure compound Shatavarin IV was procured commercially from Sigma-Aldrich Solution, Merck, Darmstadt, Germany (CAS Number: 84633-34-1) for the *in vitro* evaluation of anti-HIV-1 activity in this study.

### 2.2 Cell lines

TZM-bl cells (HeLa modified cell line; initially called JC53-bl; clone 13) were acquired from the National Institute of Health (NIH)–HIV Reagent Program and maintained in DMEM (Gibco, MA, United States) supplemented with 10% FBS (Moregate, Bulimba, QLD, Australia), HEPES (Gibco, Waltham, MA, United States), and antibiotics (Sigma-Aldrich, St. Louis, MO, United States) at 37°C in a 5% CO_2_ humidified chamber. Cells with at least 80% confluency were utilized for subsequent experiments. Peripheral blood mononuclear cells (PBMCs) were isolated from healthy individuals’ blood, collected from the discarded blood bags received from the government blood bank, using density gradient centrifugation with Histopaque (Sigma-Aldrich, St. Louis, MO, United States) and activated with PHA-P (5 μg/mL) (Sigma-Aldrich, St. Louis, MO, United States) in RPMI 1640 medium (Gibco, Waltham, MA, United States) supplemented with 10% FBS and 5 U/mL Interleukin-2 (IL-2) (Sigma-Aldrich, St. Louis, MO, United States) for growth factor support. Activated PBMCs were employed for HIV-1 stock generation and confirmation of anti-HIV-1 activity.

### 2.3 HIV-1 stock

The HIV-1_UG070_ (X4, Subtype D), a primary isolate, was obtained from NIH ARRRP, while HIV-1_VB28_ (R5, Subtype C), Indian primary isolate, was grown at the Division of Virology, ICMR-NITVAR. Virus stocks were prepared in PHA-P (5 μg/mL) activated PBMCs derived from healthy donors and quantified by HIV-1 p24 antigen detection assay (Abcam, Cambridge, United Kingdom). These virus stocks were titrated in TZM-bl cell lines, and the TCID_50_ (i.e., 50% of the tissue culture infective dose) was determined using the Spearman-Karber method.

### 2.4 Cytotoxicity by MTT and ATPlite assay

Cellular toxicity profiles of the AR extracts and Shatavarin IV were determined using the MTT assay with TZM-bl and activated PBMCs. TZM-bl cells were seeded in 96-well plates and allowed to adhere overnight, while PBMCs were seeded directly in suspension. Serial dilutions of the extracts (0.0078–1.00 mg/mL) and/or the active molecule (0.039–5.00 mg/mL) were added to the cells, and the MTT Assay was performed after 48 h of incubation. Cell viability was measured colorimetrically, and the percentage of cell viability was calculated. The assay indirectly measured cellular toxicity, and the results were expressed as CC_50_, the concentration of a product with at least 50% viable cells. Additionally, the ATPlite assay (PerkinElmer, Netherlands) was employed for the assessment of cell cytotoxicity. This assay is a highly sensitive and widely used method for determining cell viability and cytotoxicity. It is based on the quantification of adenosine triphosphate (ATP), which is present in all metabolically active cells. In this assay, cells were seeded in 96-well plates and treated with varying concentrations of AR extracts or Shatavarin IV. Following incubation, the ATPlite reagent was added to each well, and luminescence was measured using a multi-mode microplate reader (Perkin Elmer, United States). The luminescence signal was directly proportional to the amount of ATP present, allowing for the determination of cell viability. The concentration at which 50% of cell viability was inhibited (CC_50_) was calculated from dose–response curves constructed from the data obtained. Both MTT and ATPlite assays were performed in triplicates.

### 2.5 Cell associated anti-HIV-1 assay

Subtoxic concentrations of AQAR and HAAR extracts and/or bioactive constituent Shatavarin IV were tested against HIV-1 primary isolates using the cell-associated anti-HIV-1 assay. TZM-bl cells (1 × 10^4^ cells/well) were seeded in microplates prior to the assay. Pre-titrated HIV-1_VB028_ and HIV-1_UG070_ virus stocks were used to infect TZM-bl cells. Two-fold serial dilutions of the drug candidates were overlaid onto the infected cells, and luciferase activity was measured after 48 h of incubation using the Britelite plus reagent (Perkin Elmer, Waltham, MA, United States). A known reverse transcriptase inhibitor, azidothymidine or AZT, was taken as a standard drug control. Dose-dependent percent inhibition was calculated using the formula below:

$$\mathrm{Percentage}\;\mathrm{Inhibition}=\left[1-\frac{\left(Avg\;RLU_{\mathrm{Test}}-Avg\;RLU_{\mathrm{Mock}}\right)}{\left(Avg\;RLU_{VC}-Avg\;RLU_{\mathrm{Mock}}\right)}\right]\times 100$$

The effective inhibitory concentration for 50% inhibition of HIV-1 replication (EC_50_) and for 80% inhibition of HIV-1 replication (EC_80_) values were calculated. The reproducibility of results was confirmed by performing three independent assays.

### 2.6 HIV-1 p24 antigen capture assay

To confirm the safety and efficacy profiles of AQAR, HAAR, and their bioactive molecule Shatavarin IV, following the initial screening using TZM-bl cells, confirmatory experiments were conducted. These experiments employed peripheral blood mononuclear cells (PBMCs), the natural host cells of HIV. PBMCs were utilized post-activation with PHA-P (5 μg/mL) to ensure their susceptibility to HIV infection. The activated cells were cultured in RPMI supplemented with 10% FBS, 25 mM HEPES buffer, Penicillin & Streptomycin (50 U/mL & 50 mg/mL), and 10 U/mL of IL-2 as described earlier. After 48 h of activation, the cells were washed and resuspended in RPMI supplemented with 2% FBS and seeded in 96-well plates (0.2× 10^4^ cells/well) prior to infection with HIV-1_VB028_ (40 TCID_50_). The infected PBMCs were treated with non-toxic concentrations of AR extracts and/or Shatavarin IV, and the experimental controls, including mock-treated cells (Mock), virus-infected cells (VC), and cells treated with a standard drug AZT (SD), were maintained throughout the assay. The seeded microplates were incubated for 5 days, and culture supernatants were harvested by centrifugation. HIV-1 p24 antigen levels in the supernatants were quantified using an ELISA kit following the manufacturer’s instructions (Abcam, Cambridge, United Kingdom). The assay was performed in triplicates, and percent inhibition along with EC50 values were calculated. The results were compared to the reference drug AZT.

### 2.7 HIV-1 viral load assay

To determine the viral copy number, activated PBMCs were employed. These PBMCs were infected with pre-titrated HIV-1_VB028_ (40 TCID_50_) strains for 4 h under conditions similar to those mentioned above. Subsequently, post-infection, the cells underwent triple washing with RPMI medium (Gibco, MA, United States) containing 2% FBS (Moregate, Bulimba, QLD, Australia). Following this, the cells were seeded into six-well plates and suspended in RPMI containing 10% FBS media. HAAR and AQAR extracts, along with Shatavarin IV, were added. On the eleventh day post-infection, the wells were terminated, and the HIV-1 viral copy number was assessed using the Abbott Real-Time Platform (Abbott, Chicago, IL, United States). The viral load assay was performed in triplicates to ensure reliability and reproducibility, and the results were compared with the reference drug AZT.

### 2.8 HIV-1 integrase inhibition assay

The inhibitory effects of AQAR and HAAR on HIV-1 integrase (INT) activity were assessed using a commercially available HIV-1 Integrase Assay Kit (XpressBio, Frederick, MD, United States) following the manufacturer’s instructions. Briefly, a Streptavidin-coated 96-well plate was coated with a double-stranded HIV-1 LTR U5 donor substrate (DS) oligonucleotide labelled with biotin. Recombinant HIV-1 integrase protein was then loaded onto the DS DNA substrate. Following the addition of AR extracts, a new double-stranded target substrate (TS) DNA with a 3′-end alteration was introduced into the enzyme reaction. Positive controls included 1.0% Sodium Azide (provided with the kit PC) and the known HIV-1 integrase inhibitor RAL (0.48 μM – Standard Drug or SD), while the integrase enzyme provided with the kit served as the negative control (Enzyme Control or EC). The activity of HIV-1 integrase involves cleaving the last two bases from the exposed 3′ end of the HIV-1 LTR DS DNA, which then integrates into the TS DNA through a strand-transfer recombination process. The reaction products were detected calorimetrically using an HRP-labelled antibody directed against the TS 3′-end alteration, and the absorbance resulting from the HRP antibody-TMB peroxidase substrate reaction was measured at 450 nm. Three independent assays were performed to ensure accuracy and consistency of the results.

### 2.9 HIV-1 protease inhibition assay

The AR extracts were assessed for potential HIV-1 Protease inhibitory activity using an HIV-1 PR inhibitor screening Fluorometric assay kit (Abcam, Cambridge, United Kingdom). Each sample was incubated with the HIV-1 PR enzyme for 15 min at room temperature. Subsequently, the fluorescent substrate was added to the wells, and absorbance was measured (excitation/emission = 330/450 nm) using a plate reader in kinetic mode for 120 min at 37°C. The kit-supplied Enzyme Control (EC) served as the negative control, while the Inhibitor Control (PC) containing Pepstatin (1 mM) and known Protease inhibitor RTV (10 μM – SD) acted as positive controls to assess the inhibition of HIV-1 Protease activity. DMSO (1%, v/v) was utilized as the vehicle to normalize background noise. Assays were replicated three times for the reliability. The percentage inhibition of HIV-1 Protease was calculated based on the Relative Fluorescence Unit (RFU) of each test sample.

### 2.10 HIV-1 reverse transcriptase inhibition assay

The inhibitory effects of AQAR and HAAR on HIV-1 Reverse Transcriptase (RTase) were assessed using a commercially available kit (Roche, Penzberg, Germany). In this assay, the extracts at their respective EC_80_ concentrations were incubated with HIV-1 RTase and a template nucleotide mixture for 1 h. The resulting mixture was then transferred to streptavidin-coated microwell plates, where it bound to biotin, forming a complex with the DIG-labelled template primer. Subsequently, HRP-conjugated enzyme was added to each well, followed by another incubation period of 1 h. Absorbance readings were taken at wavelengths of 405 nm and 490 nm using a BioRad reader PR4100 after the addition of the substrate. Three independent assay replicates were taken into consideration for the data interpretation. AZT, a known inhibitor of HIV-1 RTase, served as a positive control (SD) in the assay.

### 2.11 Protein structure retrieval and preparation for docking simulations

The crystal structures of HIV-1 proteins, including HIV-1 Integrase (PDB: 1QS4, resolution 2.01 Å), Protease (PDB: 5KR0, resolution 1.8 Å), and Reverse Transcriptase (PDB: 3QIP, resolution 2.09 Å), were downloaded from the RCSB Protein Data Bank.^1^ These files were processed in AutoDockTools-1.5.7 to prepare them for docking simulations (Morris et al., 2009). This involved removing water molecules, other chains, and any previously docked ligands. Polar hydrogens were added, non-polar hydrogens were merged, and the energy minimization of protein residues was performed by adding Kollman charges. Any missing atoms were also repaired as necessary (Seniya et al., 2015).

### 2.12 Preparation of Shatavarin IV for docking simulations

The 2D structure of Shatavarin IV (CID: 441896), an active biomolecule found in *Asparagus racemosus*, was downloaded from the PubChem database^2^ in SDF format. The 2D structure coordinates were converted into 3D using Avogadro for docking simulation studies against HIV-1 proteins. Tautomeric and stereochemical modifications were made, and energy minimization of Shatavarin IV was performed using the Avogadro tool (Hanwell et al., 2012). The resulting 3D coordinates were saved in PDB format. Furthermore, the Shatavarin IV 3D coordinates file was processed under AutoDock4.2 to correct torsion angles and perform energy minimization by adding Gasteiger charges.

### 2.13 AutoDock program description for molecular docking

Molecular docking simulations were conducted to explore Shatavarin IV’s inhibitory potential against HIV-1 proteins. These simulations allow for the study of catalytic behaviour due to molecular interactions between the protein and the ligand Shatavarin IV. The HIV replication protein PDB and ligand 3D files were imported into AutoDock v4.2.^3^ AutoDock generated a set of ten conformation models representing the ten best models for predicting how Shatavarin IV interacts with HIV-1 Integrase, HIV-1 Protease, and HIV-1 Reverse Transcriptase. Using a Lamarckian genetic algorithm, AutoDock generated various energy values, i.e., binding energy, ligand efficiency, inhibition constant, intermolecular energy, Van der Waals, electrostatic, and total internal energy, used to analyse the relative strengths of the contacts. Binding energy, ligand efficiency, and inhibition constant are crucial indicators of the overall strength of a projected interaction assessed by AutoDock. A mass-centred grid box with a spacing of 0.375 Å was generated using the AutoGrid program and centred on active site pocket residues available in the binding pocket of HIV-1 proteins. These active site residues were identified by observing the bound proteins downloaded from the RCSB Protein database using BIOVIA Discovery Studio Visualizer v21.1.0.20298 software.^4^ These residues were later employed as the ‘flexible’ residues of the macromolecules (i.e., HIV-1 proteins) in the AutoDock experiment.

### 2.14 Interpretation and analysis of molecular docking simulation results

2D and 3D molecular interactions, along with conformational analysis, were conducted using the PMV tool within AutoDockTools v1.5.7 and the BIOVIA Discovery Studio Visualizer v21.1.0.20298 software. The binding energy was utilized to rank the ten conformations obtained in each simulation run. Conformation #1, characterized by the lowest energy, was identified as the one exhibiting the strongest binding, while conformation #10, with the highest energy, was considered to represent the weakest binding. Following established methodologies (Morris et al., 2009), we scrutinized and interpreted the energy data generated by AutoDock. According to AutoDock, the binding energy is the cumulative sum of intermolecular forces acting on the receptor-ligand complex (Equation 1) (Lin et al., 2011).

(1) $$\Delta G_{\mathrm{binding}}=\Delta G_{vdw}+\Delta G_{\mathrm{elec}}+\Delta G_{\mathrm{Hbond}}+\Delta G_{\mathrm{desolv}}+\Delta G_{\mathrm{torsional}}$$

The binding energy obtained can be compared to the calculated Gibbs free energy of the naturally occurring Shatavarin IV and HIV-1 protein interactions (Equation 2), as it essentially reflects a calculated Gibbs free energy value.

(2) $$\begin{array}{l} \mathrm{Binding Energy}=\Delta G\\ R=1.987\times 10^{-3}\mathrm{kcal}/K^{*}\mathrm{mol}\\ =\mathrm{RTlnKT}=298.15K\\ K_{a}=0.72mM=\mathrm{Binding Affinity} \end{array}$$

In the AutoDock program, the inhibition constant ( $K_{i}$ ) can be computed by first determining the binding energy (ΔG) as described earlier. Subsequently, the inhibition constant ( $K_{i}$ , μM) is calculated using the formula $K_{i}=\exp\left(\frac{\Delta G}{RT}\right)$ , where R represents the universal gas constant ( $1.985x10^{-3}\frac{\mathrm{Kcal}}{\mathrm{molK}}$ ) and T denotes the temperature (298.15 K).

### 2.15 Measurement of mitochondrial ROS

To assess intracellular reactive oxygen species (ROS) generation, particularly superoxide (O2•−), confocal imaging with MitoSOX Red molecular probe was employed, following a previously described method (Jadaun et al., 2022). Briefly, TZM-bl cells (1 × 10^5^) were seeded onto glass coverslips in a 6-well plate, infected with HIV-1_VB028,_ followed by treatment with AQAR, HAAR and Shatavarin IV for 24 h, and then incubated for 30 min in the dark with MitoSOX (10 μM). Subsequently, the cells were fixed with 3.7% paraformaldehyde and examined using a laser-scanning confocal microscope (Leica TCS SP8) after a phosphate-buffered saline (PBS) wash. A positive control comprising 0.1 mM Xanthine +0.01 U Xanthine oxidase, known ROS producers, was included. Using a standard magnification of 63 × 1.4 NA Oil objective, all confocal images were captured.

### 2.16 Measurement of cytosolic and mitosolic calcium

Intracellular calcium concentrations, both cytosolic and mitosolic, were determined using the cytosolic calcium indicator Fluo 3 AM and the mitochondria-specific calcium dye Rhod 2 AM, respectively. Confocal microscopy was utilized for qualitative identification of intracellular cytosolic and mitosolic calcium. TZM-bl cells (1 × 10^5^) were seeded onto glass coverslips in 6-well plates, infected with HIV-1_VB028_, and then treated with extracts and the pure compound Shatavarin IV for 24 h. Following a 30 min incubation in the dark, cells were stained with 5 μM Fluo 3 AM and 5 μM Rhod 2 AM using 0.1% (v/v) Pluronic® F-127 as a dispersant. After washing with PBS, cells were fixed with 3.7% paraformaldehyde in PBS. The coverslips, mounted on slides, were examined using a confocal microscope (Leica TCS SP8).

### 2.17 Measurement of mitochondrial membrane potential (Δψm)

To evaluate changes in mitochondrial membrane potential indicative of permeability alterations upon HIV-1 infection, we utilized the JC-1 probe, a fluorescent dye commonly used for this purpose (Invitrogen, Carlsbad, United States). The JC-1 probe exists in two forms: a monomeric form emitting green fluorescence and a J-aggregate form emitting red fluorescence. TZM-bl cells (1 × 10^5^) were cultured in six-well plates following HIV-1 infection and treatments with AR extracts and the bioactive molecule Shatavarin IV at their respective EC_80_ concentrations for 24 h. Subsequently, the cells were exposed to 1 mL of pre-warmed JC-1 staining solution (5 μg/mL) at 37°C for 20 min. After three washes with PBS and fixation with 3.7% paraformaldehyde in PBS, coverslips mounted on slides were analysed using a confocal microscope, as mentioned above.

### 2.18 Measurement of cellular apoptosis using caspase activity assays

The Caspase-Glo 3/7 and Caspase-Glo 9 Assay kits (Promega, WI, United States) were used to detect cellular apoptosis. These assays measure the enzymatic activity of caspases, essential regulators of programmed cell death. Caspase 9 initiates the intrinsic apoptotic pathway, while Caspase 3 and Caspase 7 execute apoptosis. The assays employ specific substrates linked to fluorophores (DEVD for Caspase 3/7 and LEHD for Caspase 9). Upon caspase activation, these substrates are cleaved, releasing fluorophores and generating a fluorescent signal. The increase in fluorescence intensity indicates caspase activity, providing a quantitative measure of apoptosis. To assess apoptosis in HIV-1 infected cells treated with AR extracts and Shatavarin IV, Caspase 3/7 and Caspase 9 activities were measured following the manufacturer’s instructions. The Caspase-Glo reagent, containing substrate and assay buffer, was added directly to the cells. After incubation, the signal was detected using a luminometer, allowing for the quantification of apoptosis in a 96-well culture plate format. Cells treated with Camptothecin (15 μm) were taken as a positive control in the experiment.

### 2.19 Statistical analysis

The collected data were analysed using GraphPad Prism 9.0 (GraphPad Software, United States) and MS Excel 2021. Each experiment was performed with a minimum of three replicates, and the mean ± standard deviation (SD) was calculated accordingly. Statistical significance was determined using a one-way ANOVA using GraphPad Prism version 9.0, comparing both mock-infected and infected experimental groups. Significance was considered at *p* < 0.05. In the results of all *in vitro* experiments, asterisks denote the level of significance, with * representing *p* < 0.05, ** *p* < 0.01, or *** *p* < 0.001.

## 3 Results

The current study sought to assess the anti-HIV-1 and antioxidant properties of both aqueous and hydroalcoholic extracts derived from *Asparagus Racemosus*, alongside its principal bioactive constituent, Shatavarin IV. These evaluations were conducted to explore their potential as anti-retroviral agents, thereby contributing to the screening process for novel therapeutic interventions.

### 3.1 Cytotoxic effects of AR extracts on TZM-bl cell line and PBMCs

**Figure 1:**
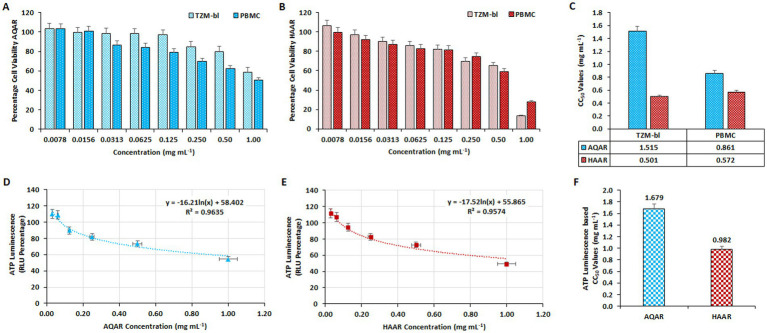
Determination of the cytotoxic concentration of *Asparagus racemosus* root extracts. The impact of varying concentrations of **(A)** Aqueous extract (AQAR) and **(B)** Hydroalcoholic extract (HAAR) on TZM-bl cells and PBMCs through MTT assay. **(C)** The average CC_50_ values of extracts derived from three separate experiments using TZM-bl and PBMC. The ATPlite Luminescence Assay to determine cell viability based on the amount of ATP present in different concentrations of **(D)** AQAR and **(E)** HAAR treated cells compared to untreated cells, based on Relative Luminescence Units (RLUs). **(F)** CC_50_ concentrations of AQAR and HAAR based on the obtained RLUs from three independent ATPlite Luminescence Assays in TZM-bl cells.

The cytotoxicity of HAAR and AQAR extracts was initially assessed on TZM-bl cell lines and PBMCs using the MTT quantitative colorimetric assay. Dose-dependent kinetics of the extracts (0.0078–1.00 mg/mL) were evaluated by plotting concentrations against the percentage of cell viability. AQAR was found to be less-toxic (<50%) to both cell lines at concentrations up to 1.0 mg/mL, whereas HAAR exhibited tolerability up to 0.50 mg/mL (Figures 1A,B). The CC_50_ values for AQAR and HAAR extracts on TZM-bl cells were determined as 1.51 mg/mL and 0.50 mg/mL, respectively. A similar trend was observed in PBMCs, with CC_50_ values of 0.86 mg/mL and 0.57 mg/mL for the aqueous and hydroalcoholic extracts of AR, respectively (Figure 1C). Additionally, the results of the MTT assays were corroborated by the ATPlite assay in TZM-bl cells (Figures 1D,E), with CC_50_ values of 1.67 mg/mL and 0.98 mg/mL obtained for AQAR and HAAR, respectively (Figure 1F). Concentrations below the determined cytotoxic levels, as indicated by the CC_50_ values, were selected for anti-HIV-1 screening.

### 3.2 Anti-HIV-1 activities of *Asparagus racemosus* extracts

**Figure 2:**
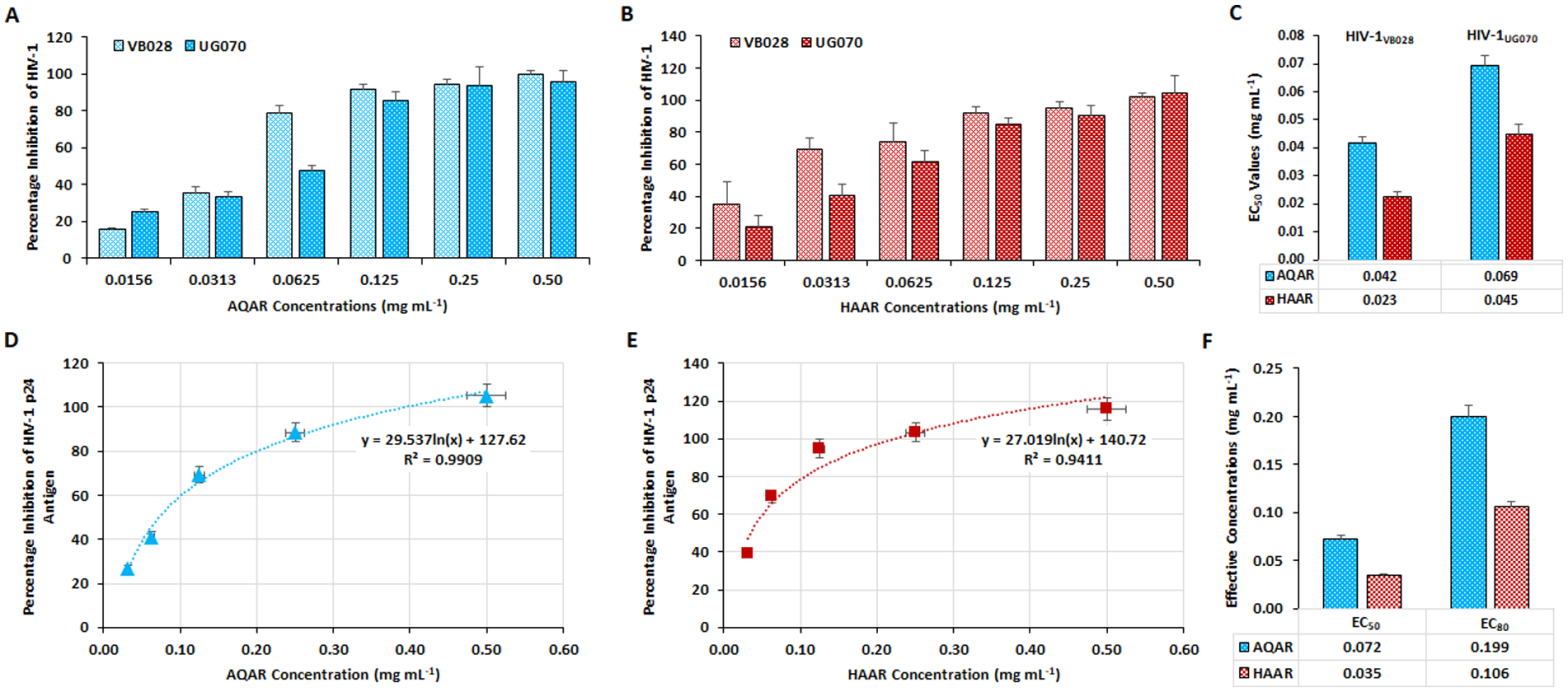
Anti-HIV-1 activity of *Asparagus racemosus* root extracts. Percentage inhibition of HIV-1 replication by **(A)** Aqueous extract (AQAR) and **(B)** Hydroalcoholic extract (HAAR) in HIV-1_VB028_ (R5 – Subtype C) and HIV-1_UG070_ (X4 – Subtype D) infected TZM-bl cells through Cell Associate assays. **(C)** The average effective concentrations (EC_50_ values) of extracts for inhibition of 50% viral infections derived from three independent experiments using TZM-bl cells. Dose-dependent kinetics of **(D)** AQAR and **(E)** HAAR treatments in HIV-1_VB028_ viral antigen – p24 inhibition in PBMCs through Cell Associated assays. **(F)** Comparison between the EC_50_ and EC_80_ values of AQAR and HAAR in HIV-1_VB028_ PBMCs.

Cell-associated assays were employed to assess the anti-HIV-1 activity of AQAR and HAAR in the TZM-bl cell line, followed by validation in PBMCs. In these assays, azidothymidine (AZT: 0.49 μM) served as the positive control (data not shown). The luciferase gene assay was utilized in TZM-bl cells to evaluate the antiviral activity of AQAR and HAAR extracts against HIV-1 infection. Separate infections with two distinct HIV-1 subtypes (HIV-1_VB028_, subtype C, and HIV-1_UG070_, subtype D) were conducted in TZM-bl cells before treatment with various doses of extracts. After 48 h of incubation, dose-dependent inhibition of HIV-1_VB028_ and HIV-1_UG070_ viral strains was observed with both extracts across different dosages (0.0156–0.50 mg/mL) (Figures 2A,B). The 50% effective concentration or EC_50_ values for AQAR and HAAR against HIV-1_VB028_ were determined to be 0.042 mg/mL, respectively (Figure 2C). For HIV-1_UG070_, the EC_50_ values were found to be 0.069 mg/mL for AQAR and 0.045 mg/mL for HAAR (Figure 2C). These results were further validated in PBMCs using the primary isolate HIV-1_VB028_, where a dose-dependent suppression of HIV-1 p24 antigen was evident (Figures 2D,E). In PBMCs, the EC_50_ values for AQAR and HAAR were found to be 0.072 mg/mL and 0.035 mg/mL, respectively (Figure 2F). Notably, AQAR and HAAR extracts exhibited 80% suppression of HIV-1 infection at non-toxic concentrations, with EC_80_ doses determined to be 0.199 mg/mL and 0.106 mg/mL, respectively (Figure 2F). The EC_80_ concentrations of the extracts were subsequently used for enzymatic and confirmatory antiviral efficacy assays against HIV-1.

**Figure 3:**
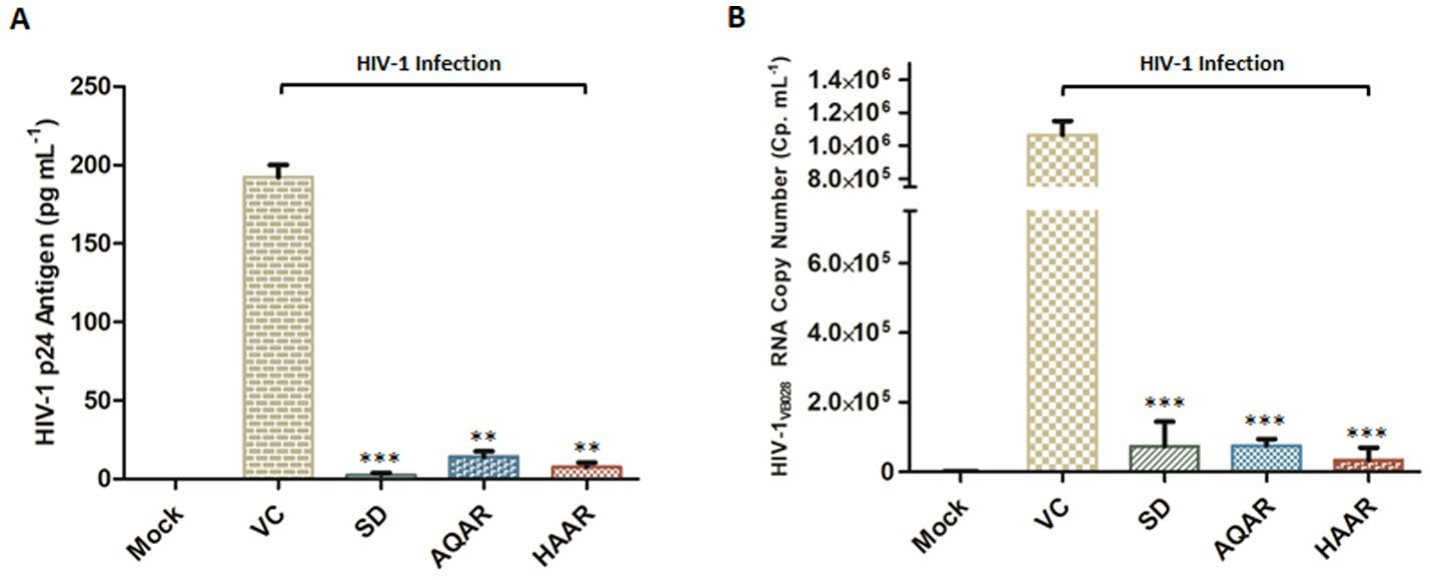
Effect of *Asparagus racemosus* root extracts on HIV-1 infection. **(A)** Level of HIV-1 p24 Antigen in presence of sub-cytotoxic concentrations (0.10 mg mL^−1^) of AQAR and HAAR treatments in HIV-1_VB028_ infected PBMCs. **(B)** Viral RNA copy numbers were assessed by Abbott m2000 platform in HIV-1_VB028_ infected PBMCs in presence of Aqueous extract (AQAR) and Hydroalcoholic extract (HAAR) of *Asparagus racemosus* root. AZT was used as standard drug control for the experiments. The results shown as the means of at least three experimental replicates plus the standard deviations were calculated and represented as the error bar. ** *p* ≤ 0.01, *** *p* < 0.001.

Additionally, extracellular p24 antigen levels (pg/mL) and HIV-1 viral copy numbers released in the culture supernatants of PBMCs infected with HIV-1 were assessed. At the eleventh-day post-infection (PID), treatment with AQAR and HAAR extracts significantly reduced p24 release by 92.6 to 96% in the supernatant, respectively (Figure 3A). Employing an automated viral load quantitative platform (Abbott m2000 System), the HIV-1 viral copy number was measured, with results depicted as RNA copies/mL (Figure 3B). Treatment with AQAR and HAAR extracts at their respective EC_80_ doses (0.19 mg/mL and 0.10 mg/mL) resulted in 29,971 copies/mL and 7,304 copies/mL, respectively, compared to the virus control (>10,00,000 copies/mL). The well-established standard drug AZT (0.49 μM) served as the positive control in both experiments.

### 3.3 *In vitro* enzymatic assays for evaluation of anti-HIV-1 activity of AR extracts

**Figure 4:**
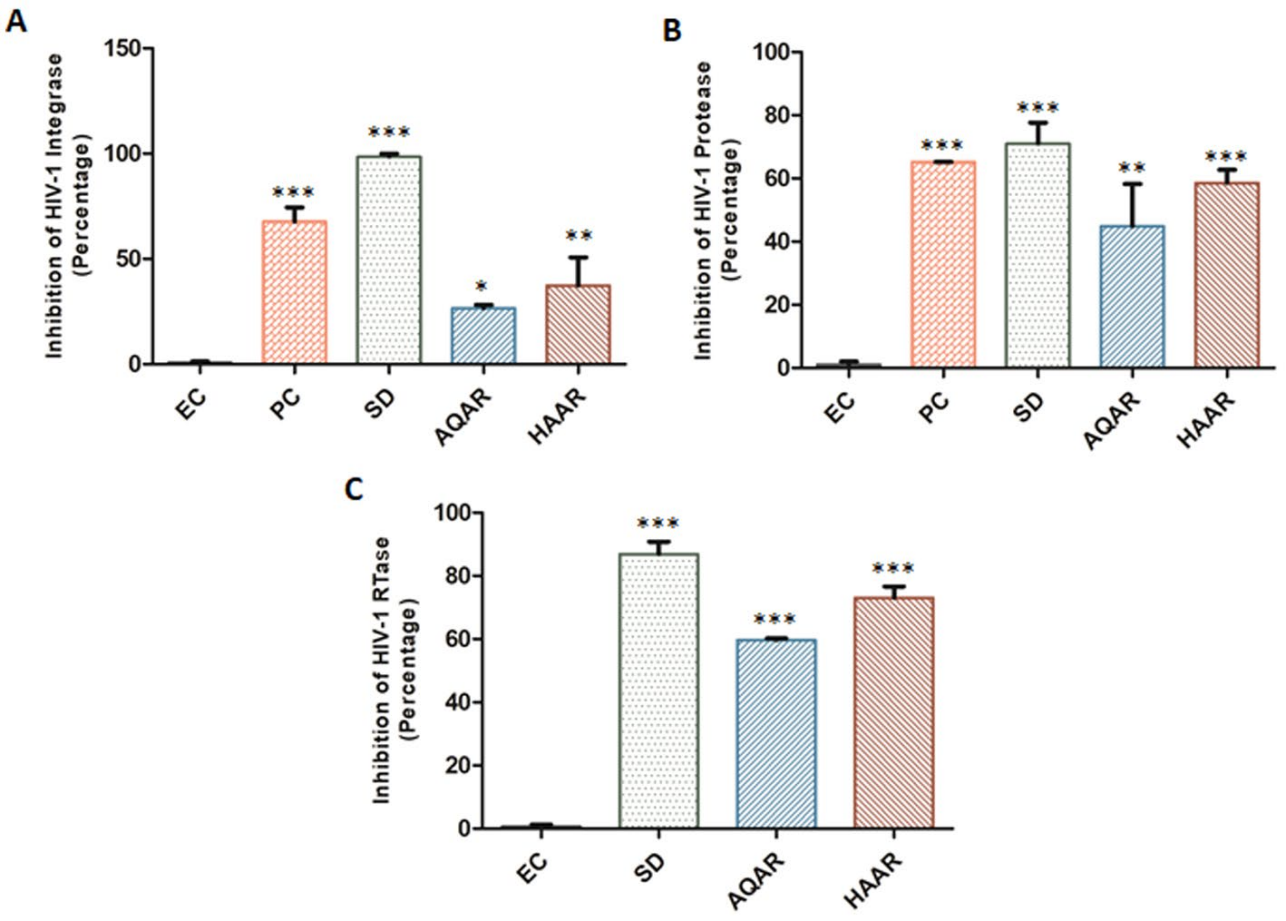
Inhibition of HIV-1 key proteins by *Asparagus racemosus* aqueous (AQAR) and hydroalcoholic (HAAR) root extracts. **(A)** Percentage inhibition of HIV-1 Integrase activities in presence of the AQAR and HAAR extracts relative to the kit provided inhibitor Enzyme as negative control (EC), Positive Control (Azide 1.0% – PC) and known Standard Drug for HIV-1 Integrase (Raltegravir 0.48 μM – SD). **(B)** The percentage of HIV-1 Protease enzyme activity suppression in the presence of extracts was compared to the kit provided Inhibitor Control or Positive Control (PC), Enzyme Control (Pepstatin 1 mM – EC), and the known Standard Drug for HIV-1 Protease (Ritonavir 10 μM – SD). **(C)** Percentage inhibition of HIV-1 RTase enzyme activity of AQAR and HAAR extracts was examined in presence of Standard HIV-1 RTase inhibitor or drug Azidothymidine (AZT 0.49 μM – SD). To normalize background fluorescence, Enzyme Control (EC) used as the negative control in each assays following the manufacturers’ instruction. The results shown as the means of at least three experimental replicates plus the standard deviations were calculated and represented as the error bar. * *p* ≤ 0.05, ** *p* ≤ 0.01, *** *p* < 0.001.

To elucidate the mechanism of action, an *in vitro* HIV-1 Integrase assay was conducted to screen both extracts at their respective EC_80_ concentrations. AQAR exhibited 26.6% inhibition, while HAAR showed 37.3% inhibition compared to the known standard drug, Raltegravir (0.48 μM; SD), which displayed 98.6% inhibition (Figure 4A). HIV-1 integrase facilitates the insertion of viral DNA into the host genome and plays a crucial role in the HIV-1 replication cycle. Notably, neither of the extracts demonstrated significant inhibition of the HIV-1 integrase protein. The kit provided Enzyme Control (EC) and 1.0% Sodium Azide (PC) were employed as the negative and positive controls, respectively.

The ability of AR extracts to suppress HIV-1 protease was evaluated using the *in vitro* kit-based assay. Results showed that AQAR extract, at a sub cytotoxic dose (0.19 mg/mL), inhibited HIV-1 protease activity by 44.9%, while HAAR (0.10 mg/mL) inhibited its activity by 58.5% (Figure 4B). When compared to Ritonavir (10 μM; SD), a well-known HIV-1 protease inhibitor, which decreased protease activity by 71.0%, both extracts exhibited moderate inhibition of HIV-1 protease. Furthermore, the assay was validated with the kit-provided positive inhibitor (PC), Pepstatin (1 mM), and Enzyme Control (EC).

Reverse transcriptase (RTase) is essential for retroviruses to replicate single-stranded viral RNA. HIV-1 RTase copies the viral RNA into double-stranded DNA, which is then integrated into the host genome. It was found that AQAR extracts inhibited HIV RTase activity by 59.7% at the EC_80_ dose, whereas HAAR extract inhibited the HIV-1 RTase enzyme by 73.0% (Figure 4C). This was compared to the well-known HIV-1 RTase inhibitor AZT (0.49 μM; SD), which inhibited the HIV-1 reverse transcriptase enzyme by 86.8%. Among the three essential replication enzymes, AR extracts exhibited the maximum reduction in reverse transcriptase enzyme activity.

### 3.4 *In silico* molecular interactions and docking analysis of Shatavarin IV with HIV-1 proteins

Molecular docking simulations utilized a key bioactive molecule, Shatavarin IV, from *Asparagus Racemosus* root extracts, identified through HR-MS using ESI+ method and Orbitrap mass analyzer, along with various HIV-1 proteins. These simulations focused on interactions between Shatavarin IV and HIV-1 proteins due to the inhibitory effects observed in *in vitro* enzymatic assays. Shatavarin IV was screened based on binding energy (Kcal/mol) and inhibition constant (*Ki*) value alongside various biomolecular interactions with HIV-1 proteins, including hydrogen bonds and hydrophobic interactions (Table 1). The inhibition constant (*Ki*) measures the strength of the interaction between a protein and a ligand, with a lower value indicating a lower dissociation and higher inhibition probability (Pandey and Verma, 2022). It is calculated as $Ki=\exp\left(\delta G/\left(R*T\right)\right)$ where *δG* is the free energy of binding, *R* is the gas constant (1.987 cal K^−1^ mol^−1^), and *T* is the temperature (298.15 K) (Morris et al., 1998). The molecular interactions demonstrate the tight binding of Shatavarin IV to HIV-1 key proteins present in the active site binding pocket, leading to structural rearrangements that achieve specific conformation and orientation, ultimately resulting in the inhibition of HIV-1 replication; hence, it is considered for further in-depth analysis. The therapeutic potential of Shatavarin IV against cancer and Parkinsonism has been documented (Mitra et al., 2012; Smita et al., 2017), but its antiviral efficacy remains unexplored. This study sheds light on stable molecular interactions between Shatavarin IV and key HIV-1 proteins (Table 1), indicating its potential as a novel antiviral agent.

**Table 1:** Molecular interactions of Shatavarin IV (CID:441896) with HIV-1 proteins.

HIV-1 Protein	Binding Energy (Kcal/mol)	Inhibition Constant (Ki)	Hydrogen Bonds	Hydrophobic Bonds	π--Alkyl Bonds	π-sigma
Integrase	−4.24	775.83	Asp64, Cys65, Thr66, Glu152, and C-H bonds with His67, Glu92, Thr66	Leu68, Gly70, Asp116, Asn155, Lys156, Lys159	Val72	His67
Protease	−7.65	12.72	Asn25, Gly27, Asp29, Asp30, Gly48 and C-H bond with Ala28, Gly49	Leu23, Val32, Lys45, Gly51, Pro79, Thr80, Ile84	Ile47, Ile50, Ile54, Leu76, Pro81, Val82	
Reverse Transcriptase	−11.48	3.86	Gln222, Lys223, Glu224	Leu100, Lys102, Lys103, Val106, Val108, Ile151, Asn155, Lys156, Tyr188, Lys220, Pro225, Phe227, Trp229, Leu234, Hid235, Pro236, Tyr318		

#### 3.4.1 Interaction with HIV-1 integrase

**Figure 5:**
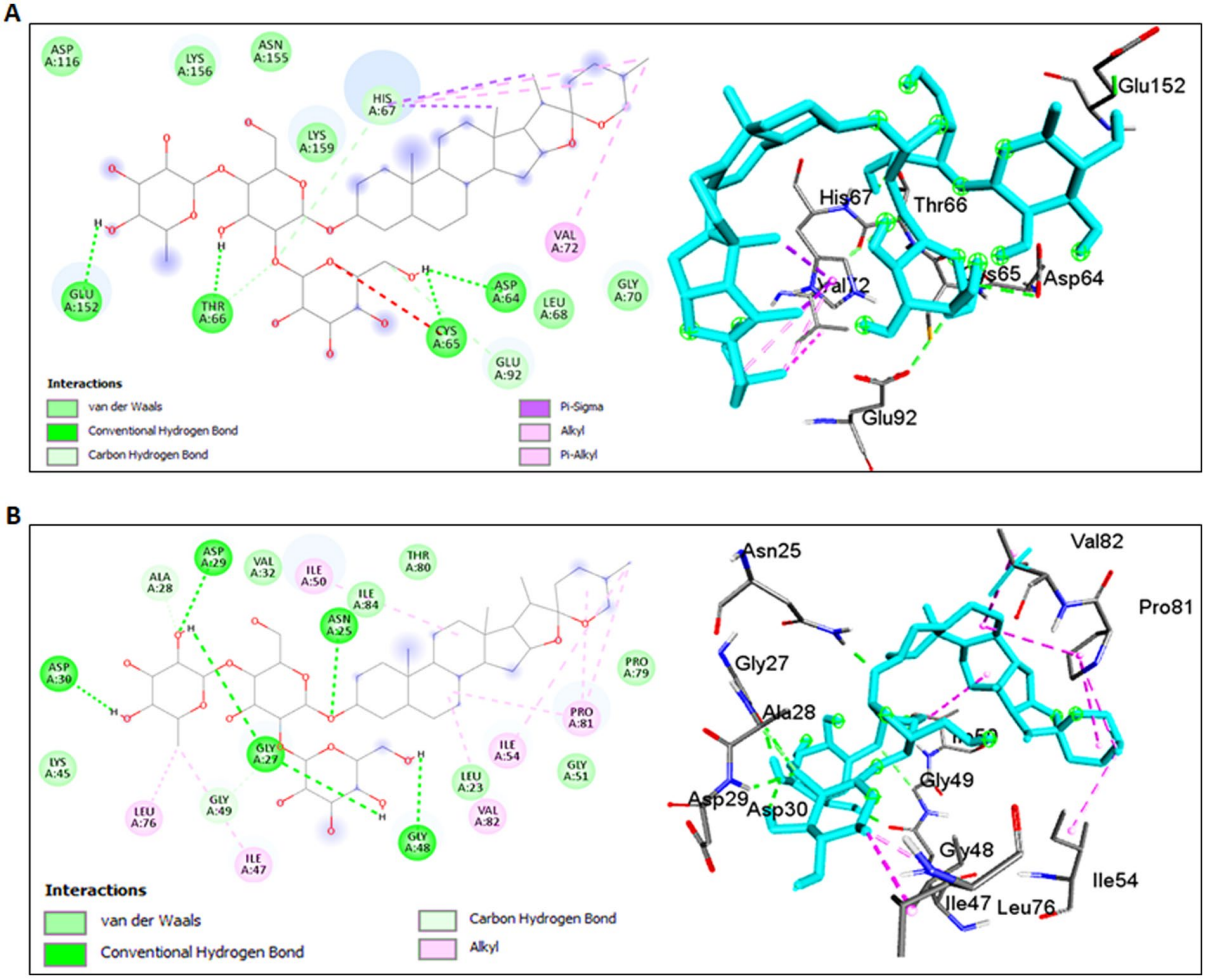
Molecular interactions of Shatavarin IV with HIV-1 enzymes. **(A)** Molecular interactions between Shatavarin IV and HIV-1 Integrase (PDB:1QS4). The left panel shows a 2D representation, while the right panel shows a 3D visualization of the docked complex, indicating the inhibition of HIV-1 infection by occupying key amino acids of HIV-1 Integrase. **(B)** Molecular docking analysis between Shatavarin IV and HIV-1 Protease (PDB:5KR0). The left panel presents a 2D representation, and the right panel displays a 3D visualization of the docked complex, illustrating the interactions of important amino acids by Shatavarin IV, resulting in structural rearrangements affecting the polymorphism in the hinge region.

Molecular docking simulations using the standard 3D structure of HIV-1 Integrase (PDB: 1QS4) revealed significant molecular rearrangements induced by Shatavarin IV. The analysis showed low binding energies of −4.24 Kcal/mol with a higher inhibition constant (*Ki*) of 775.83 μM (Table 1), suggesting moderate inhibitory effects. Shatavarin IV established multiple hydrogen bonds, C-H bonds, and hydrophobic interactions with key residues in the active binding pocket of HIV-1 Integrase, indicating its potential efficacy in inhibiting viral replication (Table 1; Figure 5A). Notably, Shatavarin IV exhibits lower binding and, therefore less inhibitory potential compared to FDA-approved HIV-1 Integrase inhibitors such as Cabotegravir, Dolutegravir, or Raltegravir (Jadaun et al., 2023a).

#### 3.4.2 Interaction with HIV-1 protease

The interaction between Shatavarin IV and HIV-1 Protease demonstrated a favourable binding energy of −7.65 Kcal/mol with *Ki* value of 12.72 μM, indicating robust structural rearrangements and association within 4 Å cut-off at the active binding pocket (Table 1). Shatavarin IV established five hydrogen bonds, two C-H bonds and seven hydrophobic bond interactions with critical residues involved in the activation of HIV-1 Protease (Table 1; Figure 5B). Given that these residues are crucial for HIV-1 Protease activation (Liu et al., 2016), their occupancy by Shatavarin IV may exert an inhibitory effect on HIV replication and maturation. Furthermore, in comparison to the FDA-approved drug Ritonavir (Jadaun et al., 2023a), Shatavarin IV exhibited the potential for more stable interactions, underscoring its efficacy as an inhibitor of HIV-1 infection.

#### 3.4.3 Interaction with HIV-1 reverse transcriptase

**Figure 6:**
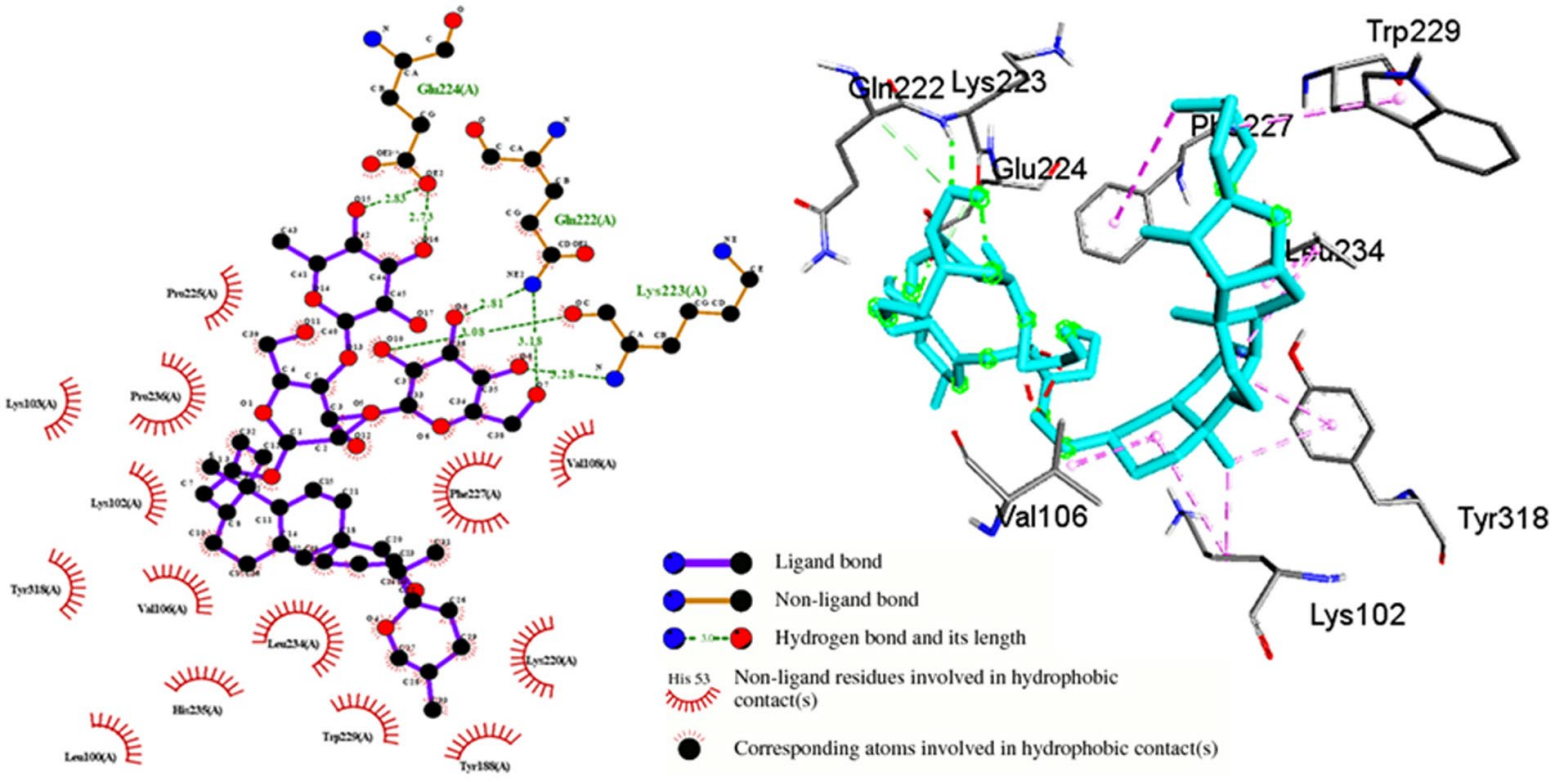
Molecular interactions between Shatavarin IV and HIV-1 reverse transcriptase (PDB:3QIP). The left panel depicts a 2D representation, while the right panel presents a 3D visualization of the docked complex. The right-handed p66 polymerase domain of HIV-1 RTase consists of thumb subdomain residues (244–322), palm subdomain residues (85–119 and 151–243), linking subdomain residues (323–427), and finger subdomain residues (1–84 and 120–150). These domains come together to form a primer-binding cleft in the palm subdomain’s β6-β10-β9 sheet, which contains polymerase active site residues. Shatavarin IV binds perfectly to the palm subdomain residues (LEU100-VAL119 and ILE151-156, TYR188, LYS220-PRO236), as shown in the figure, thereby blocking HIV-1 replication.

Shatavarin IV demonstrated a remarkably high binding energy of −11.48 Kcal/mol with a *Ki* value of 3.86 μM when interacting with HIV-1 Reverse Transcriptase (PDB:3QIP) (Table 1). The molecule formed three distinct hydrogen bonds with Gln222, Lys223, Glu224 and seventeen hydrophobic or Van der Walls interactions with Leu100, Lys102, Lys103, Val106, Val108, Ile151, Asn155, Lys156, Tyr188, Lys220, Pro225, Phe227, Trp229, Leu234, Hid235, Pro236, and Tyr318 residues within a 4 Å cut-off radius found in the active site binding pocket of HIV-1 RTase (Table 1; Figure 6). Moreover, the lowest inhibition constant value (*Ki* = 3.86 μM) indicates a lower dissociation rate with the maximum potential to inhibit HIV-1 replication. Compared to the binding energy of −7.23 Kcal/mol for the FDA-approved drug Zidovudine (Jadaun et al., 2023a), Shatavarin IV exhibited stronger interactions, indicating its robustness as a potential inhibitor of HIV-1 Reverse Transcriptase and viral replication.

### 3.5 *In vitro* assessment of toxicity profile and anti-HIV-1 effects of Shatavarin IV

**Figure 7:**
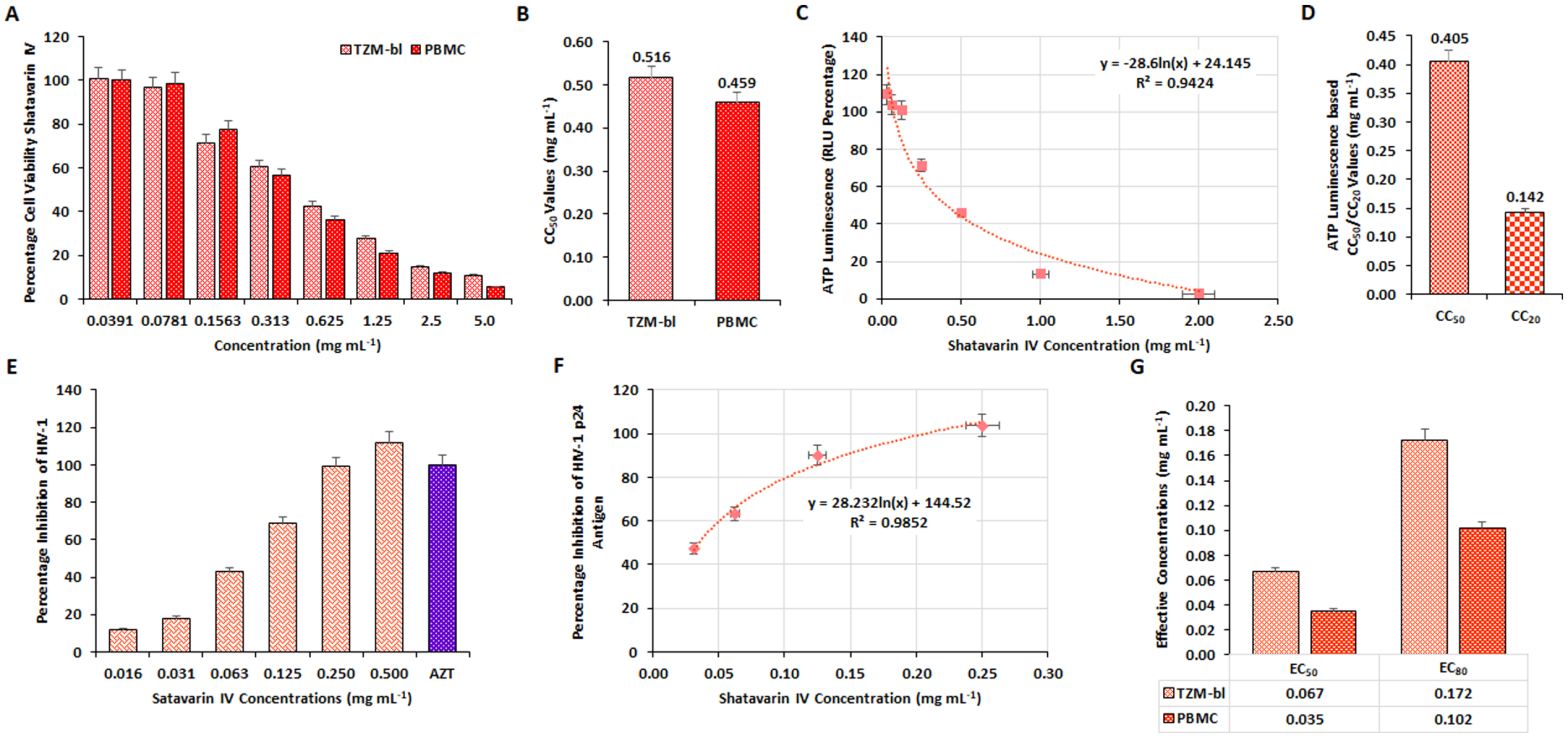
Evaluation of cytotoxic and inhibitory concentration of Shatavarin IV. **(A)** The impact of varying concentrations of Shatavarin IV on percentage cell viability were examined on TZM-bl cells and human PBMCs through MTT assay. **(B)** The average CC_50_ values derived from three separate experiments using TZM-bl and PBMC. **(C)** The ATPlite Luminescence Assay to determine cell viability based on the amount of ATP present in different concentrations of Shatavarin IV treated cells compared to untreated cells, based on Relative Luminescence Units (RLUs). **(D)** CC_50_ and CC_20_ concentrations of Shatavarin IV based on the obtained RLUs from three independent ATPlite Luminescence Assays in TZM-bl cells. The Inhibitory concentration of Shatavarin IV against HIV-1 in **(E)** TZM-bl cells and **(F)** human PBMCs were determined using luciferase gene assay in a dose-dependent kinetics. **(G)** Comparison between the EC_50_ and EC_80_ values of Shatavarin IV in HIV-1_VB028_ infected TZM-bl cells and human PBMCs.

To verify the bioactivity of Shatavarin IV, the pure compound was initially assessed for cellular viability on TZM-bl cell lines and PBMCs using the MTT quantitative colorimetric assay. Concentration-dependent effects of the extracts (0.039–5.00 mg/mL) were observed, with concentrations plotted against the percentage of cell viability (Figure 7A). The CC_50_ values were determined to be 0.516 mg/mL for TZM-bl cells and 0.419 mg/mL for PBMCs (Figure 7B). These results were further supported by ATPlite assay results in TZM-bl cells (Figure 7C), with CC_50_ and CC_20_ (cytotoxic concentration causes a 20% reduction in cell viability) values for Shatavarin IV determined as 0.405 mg/mL and 0.142 mg/mL, respectively (Figure 7D).

Concentrations ranging below the CC_50_ value (0.016–0.500 mg/mL) of Shatavarin IV were then assessed to determine its anti-HIV-1 activity using a luciferase gene assay in HIV-1_VB028_ infected TZM-bl cells, with further confirmation in PBMCs (Figures 7E,F). Shatavarin IV treatment for HIV-1 suppression yielded EC_50_ and EC_80_ values of 0.067 mg/mL and 0.172 mg/mL in TZM-bl cells, respectively, while in PBMCs, the values were 0.035 mg/mL and 0.102 mg/mL (Figure 7G).

**Figure 8:**
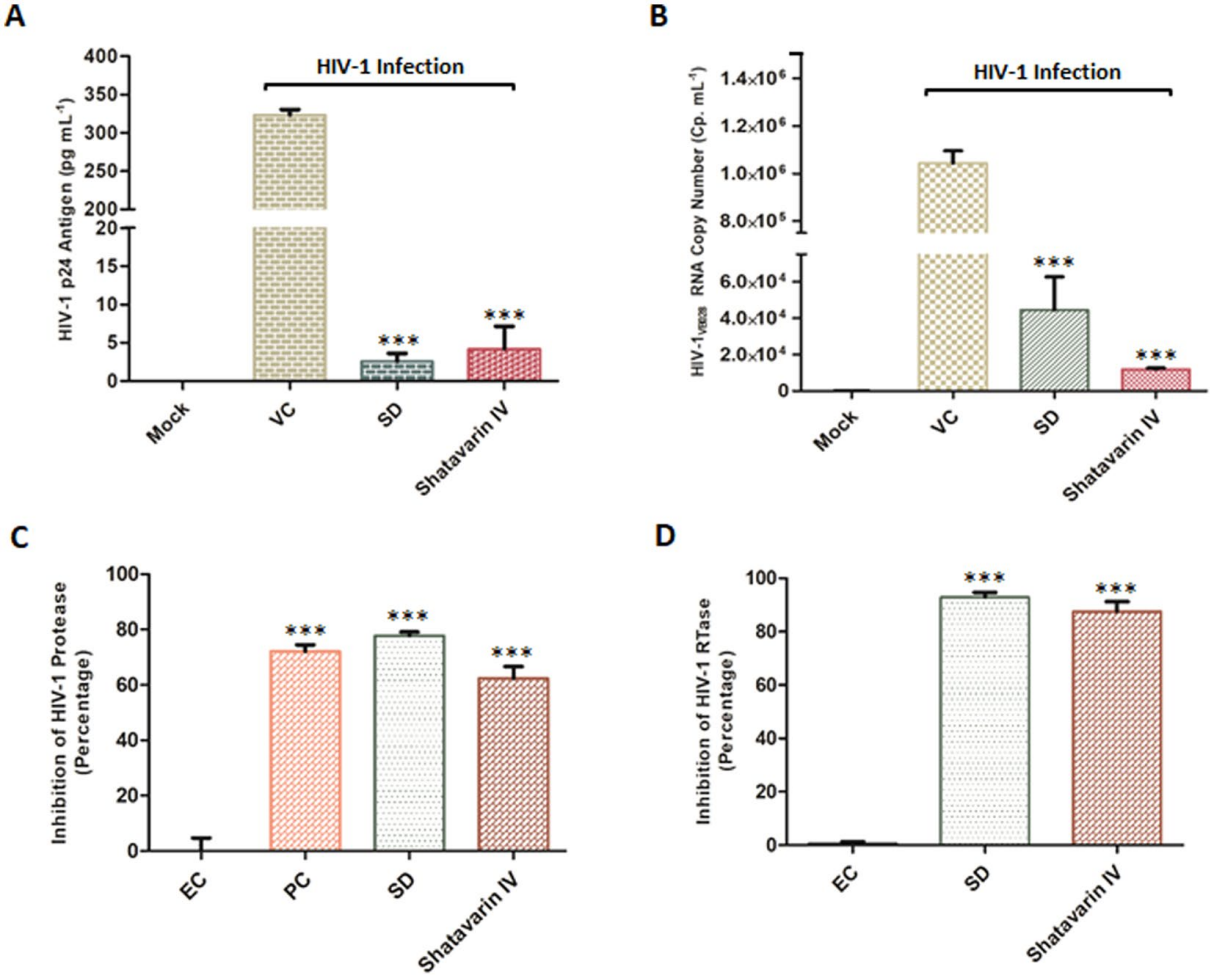
Effect of Shatavarin IV, the bioactive molecule of *Asparagus racemosus,* on HIV-1 infection. **(A)** Level of HIV-1 p24 Antigen in presence of sub-cytotoxic concentrations (0.10 mg mL^−1^) of Shatavarin IV treatments in HIV-1_VB028_ infected PBMCs. **(B)** Viral RNA copy numbers were assessed by Abbott m2000 platform in HIV-1_VB028_ infected PBMCs in presence of Shatavarin IV. AZT was used as standard drug control for the experiments. **(C)** The percentage of HIV-1 Protease enzyme activity suppression in the presence of Shatavarin IV was compared to the kit provided Inhibitor Control or Positive Control (PC), Enzyme Control (Pepstatin 1 mM – EC), and the known Standard Drug for HIV-1 Protease (Ritonavir – SD). **(D)** Percentage inhibition of HIV-1 RTase enzyme activity of Shatavarin IV was examined in presence of Standard HIV-1 RTase inhibitor or drug Azidothymidine (AZT – SD). To normalize background fluorescence, Enzyme Control (EC) used as the negative control in each assays following the manufacturers’ instruction. All results shown as the means of at least three experimental replicates plus the standard deviations were calculated and represented as the error bar. *** *p* < 0.001.

Furthermore, treatment with Shatavarin IV at a sub cytotoxic dose (0.10 mg/mL) significantly suppressed p24 antigen (pg/mL) and viral RNA levels by over 98% (98.3–98.8%) in PBMCs supernatant at 11th PID, as confirmed by extracellular HIV-1 p24 antigen (pg/mL) and HIV-1 viral load assays, highlighting its potent role as an inhibitor of HIV-1 (Figures 8A,B). Remarkably, in the HIV-1 viral load assay, Shatavarin IV-treated HIV-1 infected cells showed a count of 11,808 cp/mL, whereas the virus control exhibited >10,00,000 cp/mL (Figure 8B).

To elucidate the probable mechanism underlying the potent anti-HIV-1 activity of Shatavarin IV, we tested its effects on key HIV-1 enzymes *in vitro* to validate the molecular interaction studies. Shatavarin IV demonstrated significant inhibitory activity against both HIV-1 protease and reverse transcriptase. At a sub-cytotoxic concentration (0.10 mg/mL), Shatavarin IV inhibited HIV-1 protease activity by 62.4%, compared to the standard drug Ritonavir, which reduced protease activity by 77.8% (Figure 8C). Moreover, Shatavarin IV exhibited substantial inhibition of HIV-1 RTase, reducing its activity by 87.6%, compared to AZT, which inhibited RTase by 92.9% (Figure 8D). These results align with molecular interaction studies, where Shatavarin IV displayed strong binding affinity to the active sites of both HIV-1 PR and RTase, suggesting that its inhibitory effects arise from effective molecular interactions with these enzymes, potentially disrupting their catalytic functions. This mechanistic insight underscores Shatavarin IV’s promising role as a bioactive compound in inhibiting HIV-1 replication.

### 3.6 ROS scavenging potential of AR extracts and Shatavarin IV in HIV-1 infection

**Figure 9:**
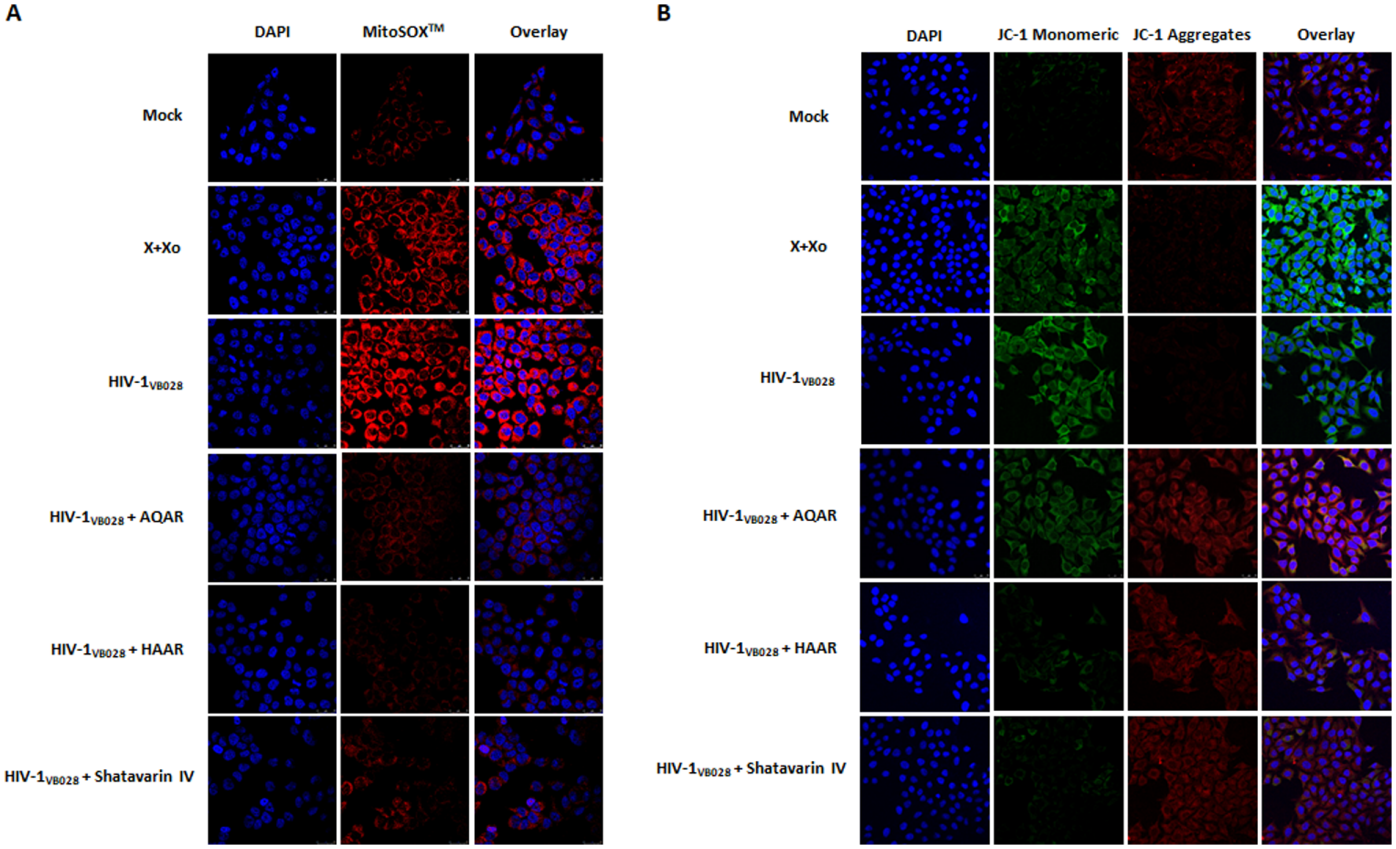
Role of *Asparagus racemosus* in mitochondrial dysfunction during HIV-1 infection. **(A)** Mitochondrial superoxide production was detected with MitoSOX™ Red Probe staining (Ex/Em 510/610 nm) in mock or HIV-1_VB028_ infected TZM-bl cells treated with AQAR, HAAR, or Shatavarin IV. Confocal microscopy images show mitochondrial superoxide (red) and nuclear DNA (blue) at 24 hpi, captured with a 63 × 1.4 NA oil objective. **(B)** Representative confocal microscopy analysis of mitochondrial membrane potential using JC-1 staining, where red fluorescence indicates the mitochondrial aggregate JC-1, while green fluorescence represents the monomeric JC-1 in TZM-bl cells. Mock or HIV-1_VB028_ infected cells treated with AR extracts or Shatavarin IV were analysed for JC-1 staining (red/green) and nuclear DNA (blue) at 24hpi. A decrease in the ratio of green (~529 nm) to red (~590 nm) fluorescence intensity, caused by HIV-1 infection or positive control, indicates mitochondrial membrane depolarization. Positive controls included 0.1 mM Xanthine +0.01 U Xanthine oxidase (X + Xo) for both experiments. Representative images are from three independent experiments.

The HIV-1 infection induces oxidative stress and mitochondrial dysfunction by elevating reactive oxygen species (ROS) levels (Ivanov et al., 2016). Dysregulation of mitochondrial calcium exacerbates ROS production and mitochondrial membrane potential alterations (Foo et al., 2022). Using the MitoSOX fluorescent probe, we assessed ROS levels in HIV-1 infected TZM-bl cells to evaluate the antioxidant activity of *Asparagus Racemosus* extracts and its key bioactive molecule, Shatavarin IV. Confocal microscopy revealed higher mitochondrial ROS content in HIV-1 infected cells compared to mock cells. Meanwhile, treatment with AR extracts and Shatavarin IV reduced fluorescence intensity in HIV-1 infected cells (Figure 9A; Supplementary Figure S2).

Changes in permeability result in a reduction in the potential of the mitochondrial membrane (Ψm), which can be assessed using the JC-1 dye molecular probe (Sivandzade et al., 2019). We evaluated Ψm changes in mock and HIV-1 infected cells in the presence or absence of AR extracts and Shatavarin IV treatment. Confocal microscopy revealed increased fluorescence intensity in HIV-1 infected cells, indicative of Ψm loss. However, treatment with AR extracts and Shatavarin IV restored Ψm levels similar to mock control cells (Figure 9B; Supplementary Figure S3).

**Figure 10:**
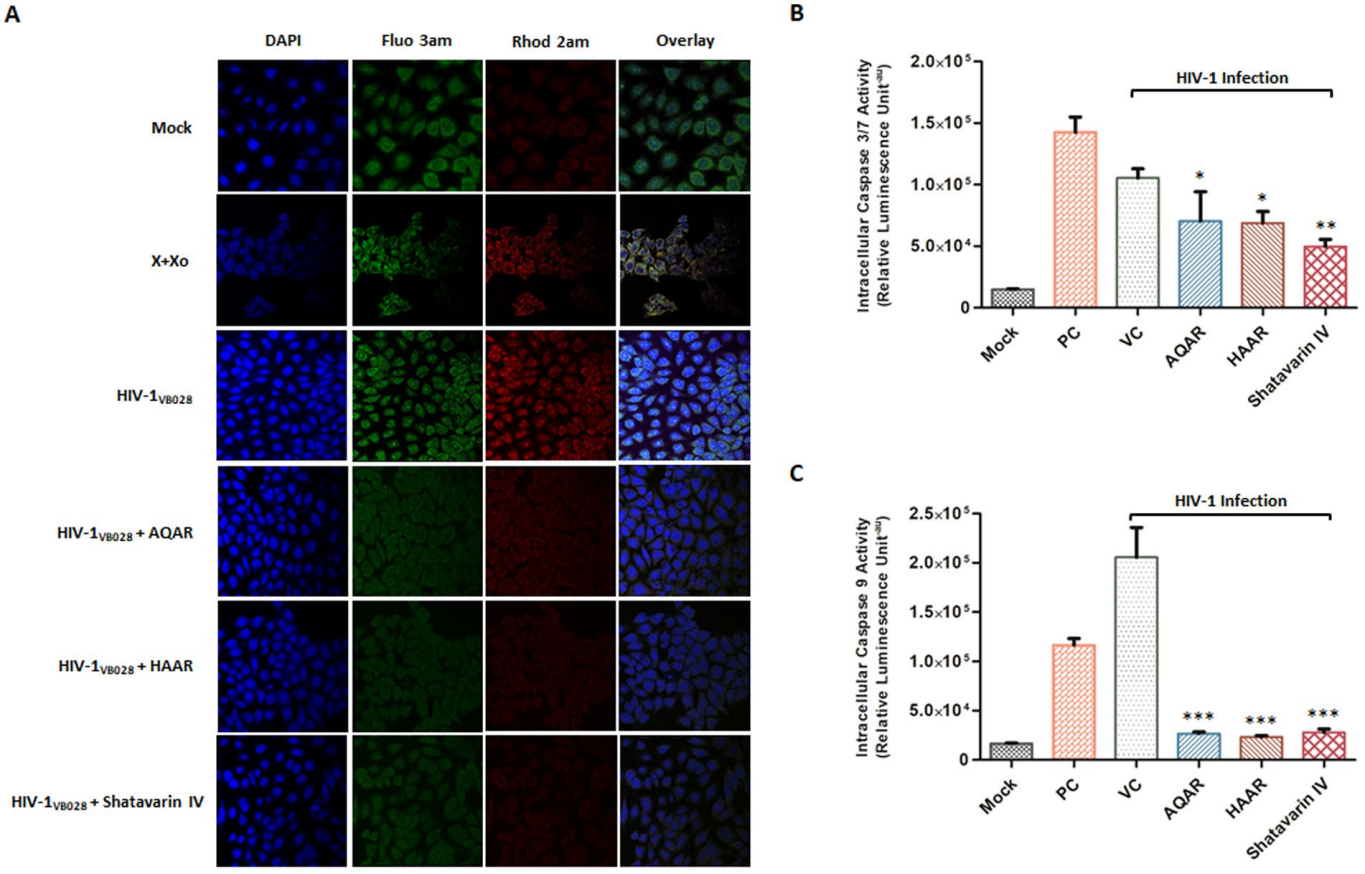
Impact of *Asparagus racemosus* on calcium imbalance and apoptosis induction during HIV-1 infection. **(A)** Fluorescent dyes Fluo-3 AM (~Ex/Em 506/525) for cytosolic calcium and Rhod-2 AM (~Ex/Em 552/581) for mitochondrial calcium were used to stain mock or HIV-1_VB028_ infected TZM-bl cells treated with AQAR, HAAR, or Shatavarin IV. Increased fluorescence of Fluo-3 AM and Rhod-2 AM in HIV-1 infected cells indicates elevated cytoplasmic and mitochondrial Ca2+ levels. Positive controls included 0.1 mM Xanthine +0.01 U Xanthine oxidase (X + Xo). Representative images are from three independent experiments. Activity of **(B)** Caspase 3/7 and **(C)** Caspase 9 at 24 hpi in HIV-1-infected and AQAR, HAAR, or Shatavarin IV treated TZM-bl cells. Cells treated with Camptothecin (15 μm) was taken as a positive control (PC). The data shown are representative of at least three independent experiments. ** *p* < 0.01 and *** *p* < 0.001.

Calcium homeostasis plays a critical role in regulating apoptotic machinery. Consequently, calcium accumulation in the mitochondria leads to the opening of the membrane permeability transition pore, resulting in a decrease in the potential of the mitochondrial membrane (Giorgi et al., 2012). To evaluate the distribution of cytosolic and mitosolic calcium in HIV-1 infected TZM-bl cells, we utilized Fluo 3 AM and Rhod 2 AM fluorescent probes. After 24 h of exposure, HIV-1 infected cells exhibited increased fluorescence intensity of both Fluo 3 AM and Rhod 2 AM, indicating elevated levels of cytosolic and mitochondrial calcium, respectively. However, treatment with AR extracts and Shatavarin IV reduced both cytosolic and mitosolic calcium levels, suggesting the restoration of mitochondrial membrane potential (Figure 10A; Supplementary Figure S4).

Caspases, members of the cysteine protease family, play a pivotal role in programmed cell death, triggered notably by HIV-1 envelope proteins (Cicala et al., 2000). Hence, we investigated the activation of caspase-3/7 and caspase-9 to assess the impact of AR extracts and Shatavarin IV in HIV-1 infection. Caspase 3/7 and Caspase 9 assays detect the enzymatic activity of these caspases, where Caspase 9 serves as an initiator caspase in the intrinsic apoptotic pathway, Caspase 3 and Caspase 7 act as effector caspases in the execution phase of apoptosis. We observed an 86% increase in Caspase-3/7 activity in HIV-1 infected cells compared to mock-infected cells (Figure 10B). However, treatment with AQAR and HAAR extracts significantly inhibited this elevated activation by 33.3 and 34.7%, respectively, compared to the virus control, while Shatavarin IV treatment exhibited a 52.9% inhibition in Caspase 3/7 activity in HIV-1 infected cells (Figure 10B). Similarly, Caspase 9 activity increased by over 92% in HIV-1 infected cells compared to mock infection (Figure 10C). However, treatment with AQAR and HAAR extracts, as well as Shatavarin IV, reduced this activation by 87, 88.7, and 86.5%, respectively, compared to the virus control (Figure 10C). Thus, extracts of *Asparagus Racemosus* and its key bioactive molecule Shatavarin IV mitigated caspase activation in HIV-1 infection. Overall, these results underscore the antiviral potential of AR and Shatavarin IV and their capability to alleviate ROS-mediated mitochondrial dysfunction.

## 4 Discussion

*Asparagus racemosus*, revered as the “Queen of herbs” in Ayurvedic medicine, holds a prominent position in traditional and global medicinal practices due to its rich therapeutic properties (Alok et al., 2013; Srivastava et al., 2018). Among its array of bioactive compounds, Shatavarin IV, a notable steroidal saponin derived from its roots, has gained attention for its diverse bioactivities (Smita et al., 2017). In light of the limitations of Antiretroviral Therapy (ART), the exploration of natural inhibitors emerges as a promising strategy (Cottura et al., 2022).

In this study, we aimed to unveil the anti-HIV-1 potential of aqueous and hydroalcoholic extracts, as well as Shatavarin IV from *Asparagus racemosus*, employing both *in vitro* and *in silico* approaches. Our investigation yielded several noteworthy findings: (1) Both aqueous and hydroalcoholic extracts exhibited a favourable safety profile alongside anti-HIV-1 activity; (2) Mechanistically, the observed inhibition targeted crucial enzymes in the HIV-1 replication cycle, including reverse transcriptase and protease; (3) Shatavarin IV, identified as a key constituent, demonstrated significant anti-HIV-1 efficacy; (4) Furthermore, both the extracts and Shatavarin IV displayed potent mitochondrial ROS scavenging activity, thereby alleviating mitochondrial dysfunction in HIV-1 infected cells. Collectively, these findings suggest that the dual action of *Asparagus racemosus* holds promise as a therapeutic strategy against HIV-1 infection.

*Asparagus racemosus* has long been revered for its safety profile, particularly during pregnancy and lactation, in line with Ayurvedic principles (Alok et al., 2013). Notably, studies in mice have demonstrated normal behaviour patterns even with higher systemic doses of the extract. Furthermore, both aqueous and hydroalcoholic extracts of *Asparagus racemosus* exhibited minimal cytotoxicity in TZM-bl cells and PBMCs, even at higher concentrations (Figure 1), corroborating previous research highlighting its safety and potential therapeutic applications. The interaction between solvents and plant constituents plays a significant role in determining both the extraction efficiency and subsequent cytotoxicity of the respective extract. Aqueous extraction primarily yields water-soluble primary metabolites, such as sugars and amino acids, which are generally less cytotoxic. In contrast, hydroalcoholic extraction facilitates the extraction of both polar and non-polar secondary metabolites, such as saponins, flavonoids, and alkaloids, which are more biologically active and potentially more cytotoxic (Jones and Kinghorn, 2006). The slightly higher cytotoxicity of the hydroalcoholic extract compared to the aqueous extract is consistent with previous studies and can be attributed to ethanol’s ability to extract a broader spectrum of bioactive compounds, some of which possess stronger cytotoxic properties (Sun et al., 2015; Prasad et al., 2024). Several *in vitro* studies and literary reports suggest that crude extracts of *Asparagus racemosus* and its constituents are rich in phytochemicals and possess anti-viral activity (Jadhav et al., 2012; Chikhale et al., 2021; Sharad and Kapur, 2021). The anti-HIV-1 activity of AR extracts observed in our study aligns with these emerging evidences of its broad antiviral potential. Recent investigations have even demonstrated its efficacy against SARS-CoV-2, highlighting its relevance in combating viral infections beyond HIV-1 (Borse et al., 2021). Our findings further contribute to this body of knowledge by elucidating the anti-retroviral potential against X4 and R5 clades of HIV-1 infection (Figures 2, 3). Additionally, we demonstrated the inhibitory effects of both aqueous and hydroalcoholic extracts on key enzymes involved in the HIV-1 replication cycle, particularly reverse transcriptase and protease (Figure 4). Notably, both extracts exhibited robust inhibition of HIV-1 RTase activity compared to moderate inhibition of HIV-1 PR activity, suggesting their potential as therapeutic agents against HIV-1 replication. These results corroborate previous findings suggesting that Ayurvedic botanicals play an important role in the enzymatic inhibitory activities of HIV-1 replication (Jadaun et al., 2023a).

Key HIV-1 replication enzymes – integrase, protease, and reverse transcriptase, are crucial targets for antiviral drugs (Engelman and Cherepanov, 2012). Molecular docking sheds light on interactions between small molecules and proteins, providing insights into their behaviour within target binding sites and essential biochemical processes. This method predicts ligand conformation, determines pose within binding sites, and assesses binding affinity (McConkey et al., 2002). Molecular docking simulations further supported the anti-HIV-1 potential of *Asparagus racemosus*, with Shatavarin IV, a prominent steroidal saponin found in its roots, establishing multiple interactions with HIV-1 RTase residues (Figure 6). These interactions suggest a mechanism by which Shatavarin IV may interfere with viral replication, highlighting its promise as an anti-HIV-1 agent. *In vitro* validation of the anti-HIV-1 activity of Shatavarin IV confirmed its efficacy through luciferase gene assays, P24 antigen capture ELISA, and quantification of HIV-1 viral copy numbers (Figures 7, 8). These findings establish Shatavarin IV as a potent inhibitor of HIV-1 replication, warranting further investigation into its therapeutic potential. To our knowledge this is the first report of anti-HIV-1 activity of Shatavarin IV as a pure compound.

Numerous investigations have unveiled that viral interactions with mitochondrial membranes and other associated components provoke heightened production of reactive oxygen species (ROS) (Dan Dunn et al., 2015; Foo et al., 2022; Purandare et al., 2023). These virus-triggered mitochondrial ROS (mtROS) subsequently facilitate viral replication by influencing host pathways and inducing covalent alterations in viral components (Foo et al., 2022; Gain et al., 2022). Ultimately, this leads to mtROS-induced apoptosis, specifically intrinsic apoptosis, which serves as a crucial viral strategy for facilitating intracellular viral replication and timely release of viral progeny. The involvement of mitochondrial dysfunction and oxidative stress play crucial roles in HIV pathogenesis, contributing to disease progression and complications (Ivanov et al., 2016; Jadaun et al., 2022). Our study observed increased ROS generation in HIV-1 infected cells, which was effectively mitigated by AR extracts and Shatavarin IV, highlighting their antioxidant properties (Figure 9). These results align with previous research demonstrating the antioxidant activity of *Asparagus racemosus* root extract, suggesting its potential in ameliorating oxidative stress-induced damage (Kongkaneramit et al., 2011). The elevated ROS levels can lead to disruption of cellular calcium homeostasis (Hempel and Trebak, 2017; Verma et al., 2021). ROS can cause the release of calcium ions from intracellular stores such as endoplasmic reticulum (ER) and mitochondria. The increased calcium levels in the cytoplasm can directly disrupt their membrane potential and impair mitochondrial respiration, leading to mitochondrial dysfunction (Shang et al., 2022). Treatment with AR extracts and Shatavarin IV reduced the effects of increased calcium levels in HIV-1-infected cells, indicating the restoration of mitochondrial membrane potential (Figure 10). This observation underscores the importance of calcium homeostasis in mitochondrial function and its modulation as a potential therapeutic strategy in HIV-1 infection. Mitochondrial dysfunction and disruption of the mitochondrial membrane integrity can lead to the release of cytochrome c into the cytoplasm through intrinsic apoptosis pathway which leads to activation of caspase-9 and subsequent activation of effector caspases (Deng et al., 2008; Li et al., 2017). Activation of caspases, particularly caspase-3/7 and caspase-9, is implicated in HIV-induced apoptosis and disease progression. Our study revealed a decrease in caspase activation in HIV-1-infected cells upon supplementation with AR extracts and Shatavarin IV, suggesting their role in mitigating intrinsic apoptosis pathway activation (Figure 10). Interestingly, we observed that at higher concentrations, AR extracts and Shatavarin IV exhibited cytotoxic effects, while at sub-cytotoxic concentrations, they demonstrated a protective effect on mitochondrial function. This dual nature of action is consistent with the behaviour of certain antioxidants, which can be beneficial at lower concentrations but potentially cytotoxic at higher levels, assumed to the prooxidant activities of antioxidants (Sotler et al., 2019; Kaźmierczak-Barańska et al., 2020).

While the findings of this study are promising, it has few limitations. The initial screening assessed the anti-HIV-1 activity of AR extracts, which inherently contain a mixture of bioactive compounds. Although this approach offers a broad range of activity, it may introduce variability in the concentration and composition of individual components and standardizing such extracts for consistent therapeutic use remains a challenge. Furthermore, the enzymatic assays did not point to specific phytochemicals, other than Shatavarin IV, responsible for the observed reductions in HIV-1 activity. However, the *in silico* analysis provided confirmation of the Shatavarin IV activity against HIV-1 enzymes, which was further validated *in vitro*. Additionally, the study did not include *in vivo* validation of the extracts and its active compound Shatavarin IV, which remains a subject for further investigation. Despite these limitations, given the constraints of current antiretroviral therapy and the significance of mitochondrial dysfunction in HIV pathogenesis, exploring alternative treatments targeting these aspects is crucial. In this study, *Asparagus racemosus* and Shatavarin IV emerge as promising candidates for novel therapeutic agents against HIV-1 infection, highlighting the importance of exploring interventions targeting this aspect of HIV pathology.

## 5 Conclusion

In conclusion, our study highlights the potential of *Asparagus racemosus* and its bioactive compound, Shatavarin IV, in combating HIV-1 infection and mitigating associated mitochondrial dysfunction. AR extracts exhibit potent anti-HIV-1 activity against diverse strains, inhibiting key enzymes crucial for viral replication. Shatavarin IV emerges as a promising anti-HIV-1 agent, demonstrating significant inhibition of reverse transcriptase activity. Moreover, both AR extracts and Shatavarin IV effectively scavenge reactive oxygen species and restore mitochondrial membrane potential, offering a therapeutic approach to alleviate HIV-1-induced mitochondrial dysfunction. The significance of these findings lies in the dual action of AR, targeting both viral replication and mitochondrial protection, making it a promising candidate in HIV-1 management. These findings also underscore the importance of exploring natural compounds like AR and Shatavarin IV as potential treatments for HIV/AIDS, warranting further research into their clinical efficacy and safety in HIV-positive individuals.

## Data Availability

The original contributions presented in the study are included in the article/Supplementary material, further inquiries can be directed to the corresponding authors.

## Footnotes

^1^http://www.rcsb.org

^2^https://pubchem.ncbi.nlm.nih.gov/

^3^https://autodock.scripps.edu

^4^https://www.3ds.com/products-services/biovia/products/molecular-modeling-simulation/biovia-discovery-studio/
