# Supplementary material for: Mitochondrial resilience and antioxidant defence against HIV-1: unveiling the power of Asparagus racemosus extracts and Shatavarin IV
Source: Front Microbiol. 2024 Oct 23;15:1475457. doi: 10.3389/fmicb.2024.1475457 (PMC11537936; doi:10.3389/fmicb.2024.1475457)
Supplement: Supplementary file 1 [file Presentation_1.pdf]

Supplementary Material

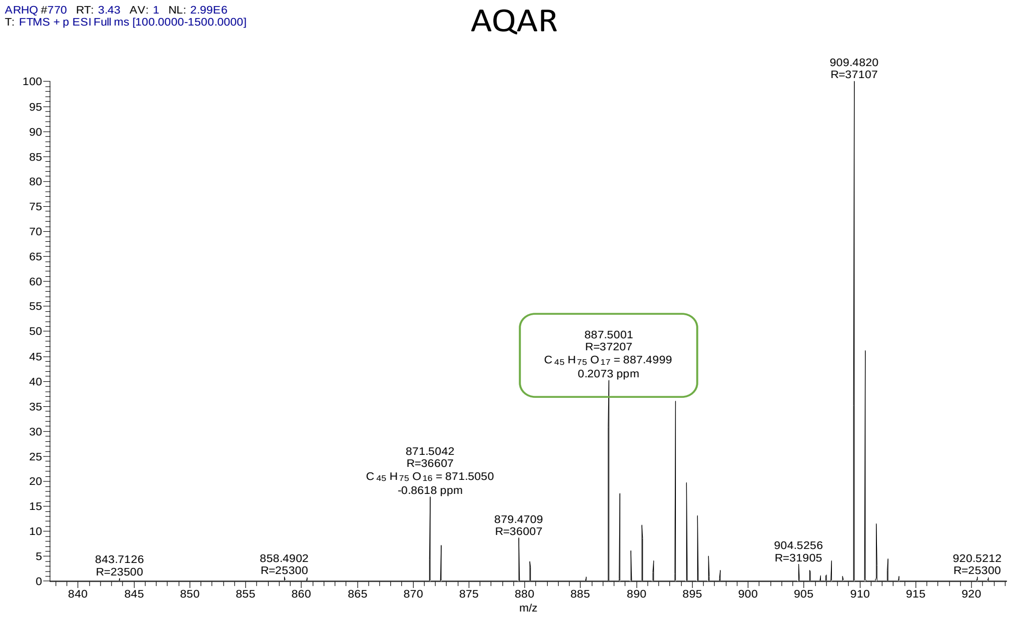

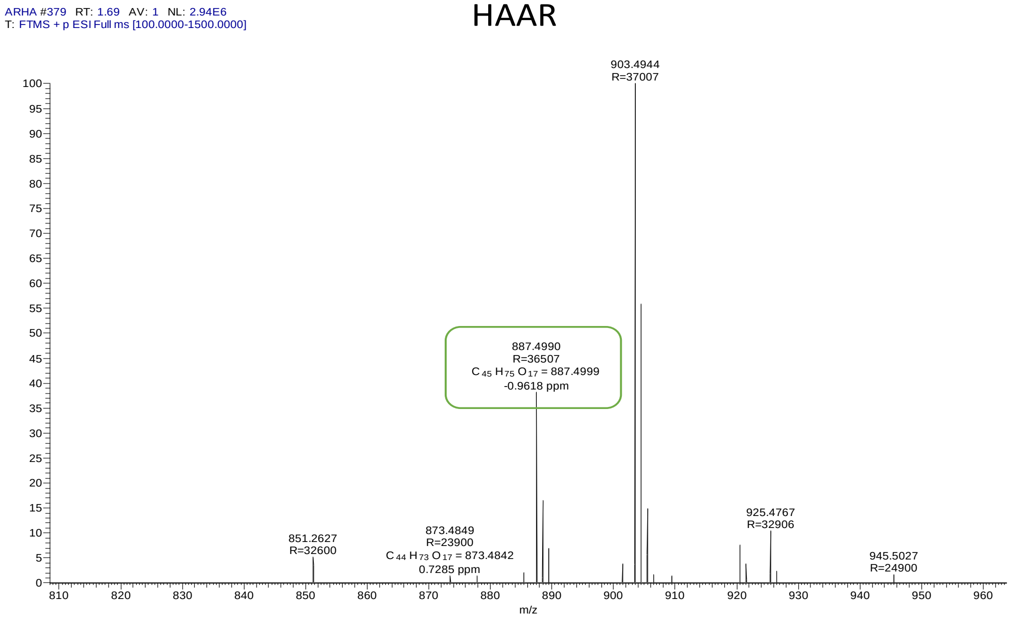

Phytoextracts	Compound	MW	Visibility
AQAR	SHATAVARIN IV	887.8	P
HAAR	SHATAVARIN IV	887.8	P

Supplementary Figure 1. High-resolution mass spectra or HR-MS for Shatavarin IV were recorded using Electrospray Ionization (ESI+) method and Orbitrap mass analyser for characterization of the Asparagus racemosus root extracts. AQAR: HRMS (ESI): m/z calcd for $C_{45}H_{74}O_{17}$ [M + H]+ 887.4990, found 887.4999 and HAAR: HRMS (ESI): m/z calcd for C45H74O17 [M + H]+ 887.5001, found 887.4999. The HR-MS data shows the presence of Shatavarin IV extract in both root extracts AQAR and HAAR.

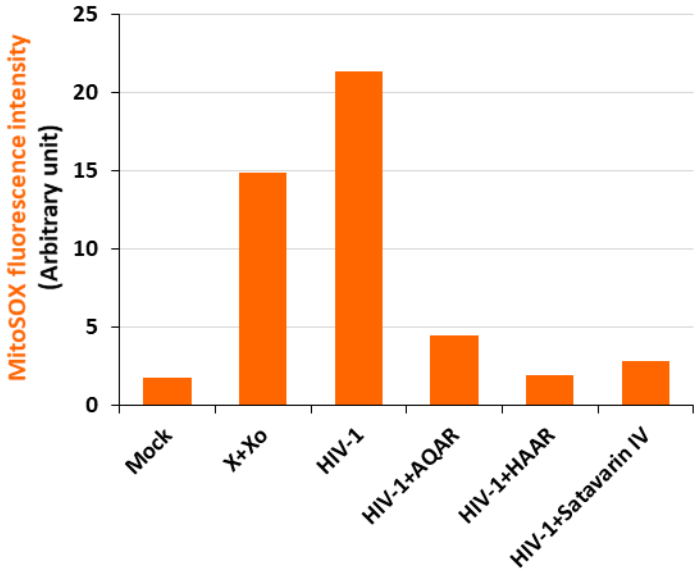

Supplementary Figure 2. Quatitative analysis of fluorescence intensity of MitoSOX levels (Figure 9A) in HIV-1 infected and AR-extracts or Shatavarin IV treated cells.

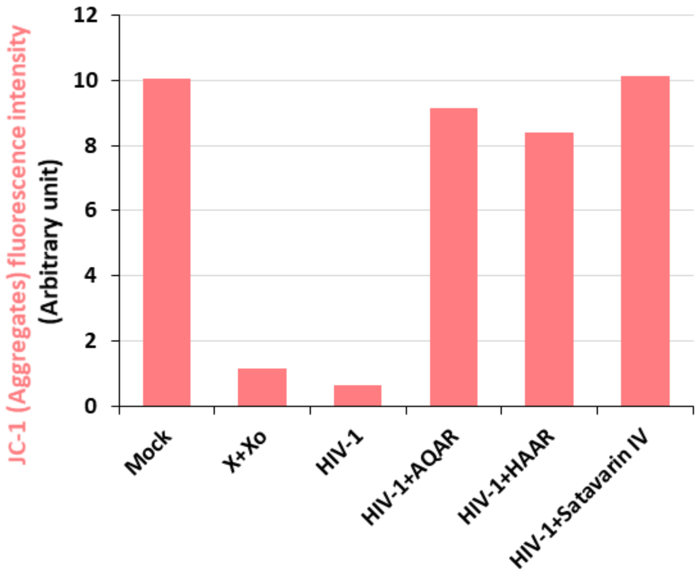

Supplementary Figure 3. Quatitative analysis of fluorescence intensity of aggregated JC-1 levels (Figure 9B) in HIV-1 infected and AR-extracts or Shatavarin IV treated cells.

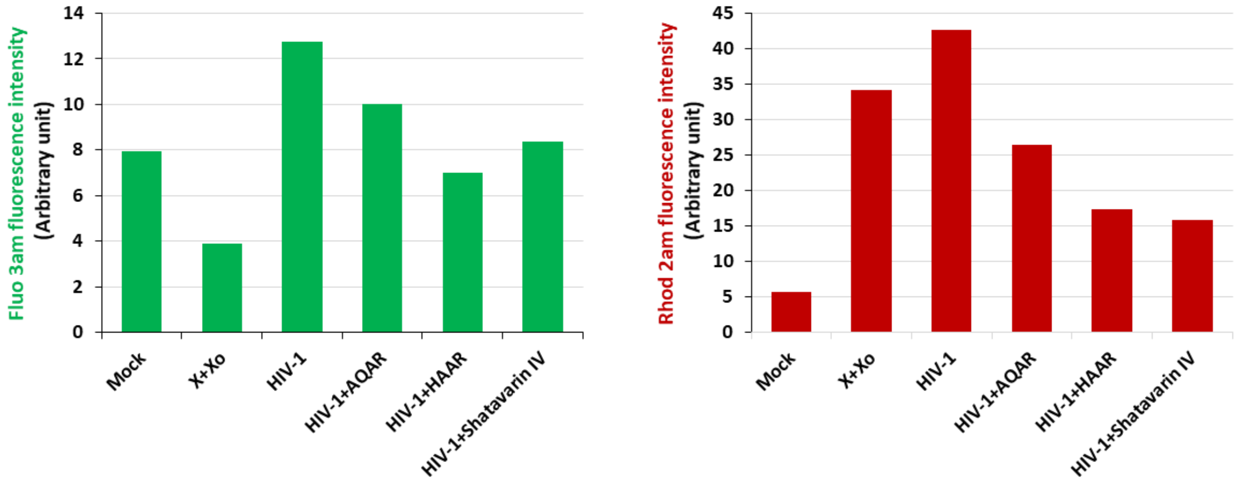

Supplementary Figure 4. Quatitative analysis of fluorescence intensity of both Fluo 3AM and Rhod 2AM indicating cytosolic and mitochondrial calcium levels (Figure 10A), respectively, in HIV-1 infected and AR-extracts or Shatavarin IV treated cells.
